# 3D Bioprinting Allows the Establishment of Long-Term 3D Culture Model for Chronic Lymphocytic Leukemia Cells

**DOI:** 10.3389/fimmu.2021.639572

**Published:** 2021-05-03

**Authors:** Francesca Vittoria Sbrana, Riccardo Pinos, Federica Barbaglio, Davide Ribezzi, Fiorella Scagnoli, Lydia Scarfò, Itedale Namro Redwan, Hector Martinez, Silvia Farè, Paolo Ghia, Cristina Scielzo

**Affiliations:** ^1^Malignant B Cells Biology and 3D Modelling Unit, Division of Experimental Oncology, IRCCS Ospedale San Raffaele, Milano, Italy; ^2^School of Medicine, Università Vita-Salute San Raffaele, Milano, Italy; ^3^Department of Chemistry, Materials and Chemical Engineering, Politecnico di Milano, Milano, Italy; ^4^B-Cell Neoplasia Unit and Strategic Research Program on CLL, Division of Experimental Oncology, IRCCS Ospedale San Raffaele, Milano, Italy; ^5^CELLINK AB, Gothenburg, Sweden

**Keywords:** chronic lymphocytic leukemia, 3D culture, bioprinting, B cell, leukemia

## Abstract

Chronic Lymphocytic Leukemia (CLL) represents the most common leukemia in the western world and remains incurable. Leukemic cells organize and interact in the lymphoid tissues, however what actually occurs in these sites has not been fully elucidated yet. Studying primary CLL cells *in vitro* is very challenging due to their short survival in culture and also to the fact that traditional two-dimensional *in vitro* models lack cellular and spatial complexity present *in vivo*. Based on these considerations, we exploited for the first time three-dimensional (3D) bioprinting to advance *in vitro* models for CLL. This technology allowed us to print CLL cells (both primary cells and cell lines) mixed with the appropriate, deeply characterized, hydrogel to generate a scaffold containing the cells, thus avoiding the direct cell seeding onto a precast 3D scaffold and paving the way to more complex models. Using this system, we were able to efficiently 3D bioprint leukemic cells and improve their viability *in vitro* that could be maintained up to 28 days. We monitored over time CLL cells viability, phenotype and gene expression, thus establishing a reproducible long-term 3D culture model for leukemia. Through RNA sequencing (RNAseq) analysis, we observed a consistent difference in gene expression profile between 2D and 3D samples, indicating a different behavior of the cells in the two different culture settings. In particular, we identified pathways upregulated in 3D, at both day 7 and 14, associated with immunoglobulins production, pro-inflammatory molecules expression, activation of cytokines/chemokines and cell-cell adhesion pathways, paralleled by a decreased production of proteins involved in DNA replication and cell division, suggesting a strong adaptation of the cells in the 3D culture. Thanks to this innovative approach, we developed a new tool that may help to better mimic the physiological 3D *in vivo* settings of leukemic cells as well as of immune cells in broader terms. This will allow for a more reliable study of the molecular and cellular interactions occurring in normal and neoplastic conditions *in vivo*, and could also be exploited for clinical purposes to test individual responses to different drugs.

## Introduction

Chronic Lymphocytic Leukemia (CLL) is the most common leukemia among adults in the Western World and it is characterized by the relentless accumulation of mature monoclonal B lymphocytes with a specific immunophenotype, positive for CD19 and CD5, along with CD23 (1). CLL is considered a dynamic and heterogeneous disease, where leukemic cells traffic and home in the peripheral blood (PB), bone marrow (BM) and secondary lymphoid tissues, such as lymph nodes (LNs) and spleen (SP) (2–5). Despite the contribution of an increasing number of studies, not only CLL is still incurable but also the underlying pathogenic mechanisms still need to be fully elucidated. In particular, mechanisms orchestrating the trafficking of the leukemic cells between the PB and the lymphoid tissues, where they organize and interact with a supportive microenvironment, have not been fully explained yet (6). Leukemic cells in the tissues establish a crosstalk with the cells from the microenvironment, which strongly support their survival and proliferation through direct contact and the secretion of specific stimuli (7, 8). Recently, Primo et al. (9) demonstrated that primary CLL B cells increase their survival and proliferation rate *in vitro* when co-cultured with stromal cells and in the presence of specific factors, such as CpG and IL2, thereby resembling the extracellular tissue microenvironment. Indeed, one of the biggest challenges in studying primary CLL cells alone *in vitro* originates from the inability to maintain their viability for a long time without the addition of exogenous stimuli that inevitably affect the function and behavior of the cells (10). A reason could be that traditional two-dimensional (2D) cultures, commonly utilized for *in vitro* studies, lack the complexity of the spatial cellular organization taking place in the tissues, providing a simplified overview of tumor biology. In addition, animal models show many limitations in particular being expensive, time consuming and not adequately reproducing all features of human tumors (11). As a consequence, it has become evident that innovative approaches are necessary to potentially overcome 2D culture-systems limitations, thus providing a better way to mimic *in vitro* what actually occurs *in vivo* (12, 13). Interestingly, over the last few years, three-dimensional (3D) culture systems have been largely implemented. The term “3D culture” refers to a 3D system in which cells can survive, proliferate, migrate, communicate and behave in a more realistic environment from a spatial point of view, and are no longer cultured on a 2D plastic or glass surface (14). In the most recent years, *in vitro* 3D models have been developed to recapitulate specialized microenvironments, such as lymphoid tissues, by integrating advanced biomaterials and microfluidics. This allowed elucidating new regulatory mechanisms and potential therapeutic targets that could have not otherwise been studied in conventional 2D cultures (15). Several 3D systems have also been applied to the study of different B cell malignancies; however, this has only recently been used for CLL and with rather limited attempts (13). In particular, we recently demonstrated the advantages of co-culturing CLL cells with bone-marrow stromal cells seeded on a 3D scaffold to study their response to targeted therapy *in vitro* (16) and, in parallel, we realized the need for exploring additional 3D culture systems to allow the growth of primary CLL cells alone as well as to improve the reproducibility of the cell seeding. Lately, relevant technological advancements have been achieved and have started being applied in biomedicine. One of the most striking is the implementation of 3D bioprinting in biomedical research, which, to date, is considered a very promising approach to generate complex and advanced 3D *in vitro* models (17, 18). Specifically, 3D bioprinting is an additive manufacturing technique in which cells are encapsulated (avoiding cell seeding limitations) within a “bioink” that ideally mimics the native extracellular matrix (ECM), and are subsequently deposited in a layer-by-layer process to a previously defined geometry (19).

In the present work, we tested for the first time whether 3D bioprinting could be applied in our system and could therefore advance *in vitro* models for CLL. We successfully evaluated CLL cells for printability, optimized the printing strategy and set-up the protocols to perform the analysis. Our results demonstrate that we can efficiently 3D bioprint primary CLL cells and improve their viability without the addition of exogenous stimuli and/or stromal cells. We can maintain and study in-culture 3D bioprinted CLL cells for up to 28 days, thus establishing an innovative and reproducible long-term 3D culture model for leukemia cells.

## Materials and Methods

### Human Ethics Statement

Patients with CLL were diagnosed according to the updated National Cancer Institute Working Group (NCIWG) guidelines (20). Peripheral blood (PB) samples were obtained after informed consent from patients who were untreated or off treatment for at least 6 months. The study was approved by the Ospedale San Raffaele (OSR) ethics committee under the protocol VIVI-CLL entitled: “*In vivo* and *in vitro* characterization on CLL”. Clinical and biological characteristics of patients with CLL who provided samples for the experiments are reported in **Supplementary Table 1**.

### Cell Culture and Human Primary Samples Purification

MEC1 cell line (21) was obtained from Deutsche Sammlung von Mikroorganismen und Zellkulturen GmbH (DSMZ, Braunschweig, Germany) and was recently genotyped as following: 10 ng of DNA from MEC1 cells was purified with QiAmp DNA Mini Kit (Qiagen, Düsseldorf, Germany) and amplified through PCR with GenePrint ^®^ 10 System (Qiagen, Düsseldorf, Germany) and sold Eurofins Genomics Standard FLA Service to perform genotyping. Data was analyzed with DSMZ Online STR Analysis. We confirmed the identity of the cell line analyzed. MEC1 cells were cultured in RPMI 1640 medium (EuroClone, Pero, Italy) supplemented with 10% (v/v) Fetal Bovine Serum (FBS) and 15 mg/ml Gentamicin (complete RPMI) at 37°C and 5% CO_2_. Leukemic CD19 cells were negatively selected from fresh peripheral blood using the RosetteSep B-lymphocyte enrichment kit according to the manufacturer protocol (StemCellTechnologies, Vancouver, Canada). Then, the Lymphoprep™ reagent (StemCellTechnologies, Vancouver, Canada) is added to the sample and centrifuged at 2000RPM, 20 minutes. After washing twice with PBS 1500RPM, 5 minutes, the cells are ready to use.

The purity of all preparations was always higher than 99%, and the cells co-expressed CD19 and CD5 on their surfaces as assayed by flow cytometry (Navios Flow Cytometer; Beckman Coulter); preparations were virtually devoid of natural killer (NK) cells, T lymphocytes, and monocytes.

### Bioink Preparation and 3D Hydrogel Scaffold Fabrication

MEC1 (21), MEC-GFP (22) or leukemic primary cells were counted, centrifuged at 1500RPM for 5 minutes, resuspended in 1:10 medium:hydrogel ratio, then gently mixed with CELLINK Bioink, CELLINK RGD10, CELLINK Laminink111, CELLINK Laminink411 or CELLINK Laminink521 hydrogels (CELLINK AB, Gothenburg, Sweden) using two luer lock syringes. We virtually calculated the number of cells potentially present in a tissue with the dimension of the printed scaffold (5x5x1mm^3^). We calculated the theoretical volume of a lymphoid cell, considering it as a sphere $\left(\frac{4}{3}\pi \;r^{3},median\;cell\;radius≃5\mu m\right)$ and the volume of the scaffold, considering it as a rectangular parallelepiped (LxLxH). We approximately estimated that to entirely fill the scaffold we should need about ≃50x10^6^ cells for scaffold, alias ≃50µl hydrogel, (n°cells=scaffold volume/cell volume). Following an experiment in which we used decreasing concentration of cells, we established a final optimal concentration ranging from 5 to 10x10^6^cells/100µl for the cell lines, and from 15 to 20x10^6^ cells/100µl for primary cells (data not shown).

The bioink mixed with the cells was then loaded in a cartridge and placed in the Bio X 3D bioprinter (CELLINK AB, Gothenburg, Sweden). The 3D scaffolds (5x5x1mm^3^) were designed with Fusion360 (Autodesk). The slicing process was directly made exploiting the Bio X slicer software, using a rectilinear pattern with 30% infill density. The Bio X was equipped with a 25G (250µm) nozzle and the layer height was set at 0.25mm. The pressure applied to the 3D bioprinting process is hydrogel/cells-dependent, a range of values around 11-14 kPa was used. All the settings of the printing process were uploaded on Bioverse (https://bioverse.com/). Printing of 3D scaffolds was directly performed in 12-well plates at 7mm/s deposition speed. The constructs were crosslinked with 50mM CaCl_2_ (CELLINK AB, Gothenburg, Sweden) for 4 minutes at room temperature and washed once with Hank’s Balanced Salt Solution (HBSS, EuroClone, Pero, Italy), according to the manufacturer protocol (CELLINK AB, Gothenburg, Sweden). RPMI complete medium (EuroClone, Pero, Italy) was added to MEC1 and MEC-GFP-laden scaffolds while CLL primary cells-laden scaffolds were added with DMEM high glucose (EuroClone, Pero, Italy) supplemented with 10% Human Serum (EuroClone, Pero, Italy) and 1% Penicillin/Streptomycin (Lonza, Basel, Switzerland), since CLL primary cells viability was found to be improved in the just-mentioned conditions from previous *in vitro* tests (data not shown). The medium was changed within 30 minutes after the printing, before the culture was placed in an incubator at 37°C, 5% CO_2_.

### Compressive Mechanical Properties

The compressive mechanical properties of 3D printed hydrogels under investigation was tested by Dynamic Mechanical Analyzer (DMA Q800, TA Instruments) with or without loaded cells. Scaffold hydrogels (n = 5) were prepared by printing of Cellink Bioink and Cellink Laminink 411 with an air pressure ranging from 11 to 14 kPa, with a deposition speed of 7 mm/s. All scaffolds were plotted with cylindrical geometry (2:3 height:diameter ratio) and crosslinked in 50mM CaCl_2_ (CELLINK AB, Gothenburg, Sweden) for 4 minutes. Tests were performed at room temperature, applying a 0,001 N preload. Each test consisted of a loading run (strain ramp = - 2,5% min^-1^ down to - 30%) followed by the unloading run (strain ramp = + 5% min^-1^ up to + 1%). The stress-strain curves were elaborated and the following mechanical parameters were considered: elastic modulus (E, considered as the slope of the regression curve in the 0-5% strain range), stiffness (K, as the slope of the regression curve in the 25-30% strain range), the maximum stress σ_max_ (corresponding to the maximum strain, i.e. ϵ = 30%), and the residual strain ϵ_res_ (corresponding to the unrecovered strain at the end of the unloading run). Rheology data of the hydrogels used (CELLINK AB, Gothenburg, Sweden) are shown in **Supplementary Figure 1**.

### Live/Dead Assay

3D bioprinted cells viability was assessed overtime by using the LIVE/DEAD^®^ Cell Imaging Kit (Thermo Fisher Scientific, Massachusetts, USA), which allows for the visualization of live (green) and dead (red) cells. The scaffolds were washed one time (30 min) with DMEM without serum (Thermo Fisher Scientific, Massachusetts, USA) and Live/Dead reagent was added in a 1:3 ratio. After 1 hour of incubation at 37°C, 5% CO_2_ the constructs were washed one time with DMEM without serum and observed with the AXIO Observer Z1 fluorescent microscope using FITC and TRITC filters, through Volocity Acquisition software, and then processed using FIJI (ImageJ) software. A grid was drawn on both the fitc-live and tritc-dead images, and live and dead cells, respectively, lying in the same fields were manually counted. Then, the percentage of live and dead cells on the total count was performed as follows: living and dead cells, respectively, were divided by the total number (live + dead) of counted cells and multiplied by 100.

### Alamar Blue Assay

The Alamar blue^®^ assay (Thermo Fisher Scientific, Massachusetts, USA) was performed on the same 3D scaffolds over time (up to 28 days of culture), in order to minimize intra-experiment replicate variability. The reagent was mixed with the appropriate medium (RPMI 1640 or DMEM complete medium) in a 1:10 ratio, respectively; then 1mL of the mix was added to each well. As assay blank, RPMI or DMEM complete medium with a 3D bioprinted scaffold without cells was used. After 4h 30’ of incubation at 37°C, 5% CO_2_, 100µl of the mix were collected and transferred to a 96-well white plate and the fluorescence values were read using the Victor spectrophotometer. The scaffolds were then washed once and placed in the proper medium for subsequent analyses.

For 2D cultures, the manufacturer’s instructions were followed. Briefly, the amount of medium per well was measured and mixed in a 1:10 ratio with the Alamar blue^®^ reagent. After 4h 30’ of incubation at 37°C, 5% CO_2_, the cells were centrifuged at 2000RPM, 5 minutes and then 100µl of the supernatant were collected and transferred to a 96-well white plate to measure fluorescence values using the Victor spectrophotometer.

### RNA Extraction and Real-Time PCR

RNA extraction was performed overtime (day 0-7-14-21-28 for 3D samples, n=16; day 0-3-7-10-14 for 2D samples, n = 16), according to the manufacturer’s instructions, using TRIzol reagent (Ambion) for 3D bioprinted scaffolds and ReliaPrep RNA Cell Miniprep System^®^ (Promega, Madison, USA) for 2D cell lines and primary samples. In general, 3D bioprinted constructs were smashed and chloroform added. The RNA is then collected after consecutive centrifugation steps and isopropanol/ethanol washes and resuspended in a variable amount of nuclease-free water. RNA from 2D cell lines and primary samples is obtained by isopropanol/DNAse solution washes and centrifugation steps. Lastly, the RNA is resuspended in nuclease-free water. cDNA was synthesized according to the manufacturer’s protocol using the RevertAid^®^ H Minus First Strand DNA Synthesis kit (Thermo Fisher Scientific, Massachusetts, USA). RT-qPCR analysis was performed using Titan HotTaq Probe qPCR mix (BioAtlas) in an ABI7900 Thermal Cycler instrument (Applied Biosystem, Foster City, USA). The analysis was performed in triplicate. Quantification of *BAX*, *BCL2, AICDA, SELL, CXCR3, CCL22, HCLS1, PIM3, MYC* transcripts (Applied Biosystem probes) was performed according to the Ct method (23), using *GAPDH/YWHAZ* as the housekeeping gene.

### RNAseq Analysis

After performing RNA extraction as described above, 3D bioprinted cells were further treated with DNase I (Thermo Fisher Scientific, Massachusetts, USA) and Ambion™ RNase Inhibitor (Thermo Fisher Scientific, Massachusetts, USA) for 15 minutes at 37°C, and eventually a second RNA extraction was performed by using ReliaPrep RNA Cell Miniprep System^®^ (Promega, Madison, USA). RNA quality was confirmed with a 2100 Bioanalyzer (Agilent), and all samples had RIN (RNA Integrity Number) greater than 7. We exploited the SMQRT-Seq^®^ v4 Ultra^®^ Low Input RNA (TaKaRa) protocol, to generate the next-generation sequencing (NGS) libraries starting from 2ng of RNA. Libraries were barcoded, pooled and sequenced on an Illumina Nova-Seq 6000 sequencing system (Illumina, San Diego, USA), in SR (single read) mode, with reads 100nt long. We estimated to obtain 80 million single-end reads per sample on average.

Reads were trimmed using Trimmomatic, version 0.39, in order to remove adapters and to exclude low-quality reads from the analysis. The remaining reads were then aligned to the reference genome GRCh38, GENCODE release 31, using STAR aligner, version 2.5.3a. FeatureCounts (v 1.6.4) was used to assign reads to the corresponding genes. Only genes with a CPM (Counts per million) value higher than 1 in at least three samples were retained. Gene expression read counts were exported and analyzed in R environment (v. 3.6.2) to identify differentially expressed genes (DEGs), using the DESeq Bioconductor library (24). p-values were adjusted using a threshold for false discovery rate (FDR) ≤ 0.05 (25). Using the 500 most variable genes in terms of RPKM (counts per million reads normalized on library sizes and gene lengths), we performed Principal Component Analysis (prcomp function in R) and clustering analysis *via* heatmap (pheatmap R library). The RNAseq data, including raw sequence files, have been submitted to NCBI’s Gene Expression Omnibus and are accessible through the GEO series accession number GSE163977.

Go to: https://www.ncbi.nlm.nih.gov/geo/query/acc.cgi?acc=GSE163977.

### Flow Cytometry

3D bioprinted scaffolds were smashed with 500µl of dissolution buffer (26), passed through a 30µm CellTrics filter (Sysmex, Kobe, Japan) to flow cytometer tubes, and eventually stained for 25 minutes RT for the following antibodies: CD5 PC5 (Beckman Coulter, Brea, USA), CD19 PC7 (Beckman Coulter, Brea, USA), and IgM PE (Miltenyi, Bergisch Gladbach, Germany). After washing with PBS 1500RPM, 5 minutes, cells were analyzed on Navios Flow Cytometer (Beckman Coulter, Brea, USA). Analyses were performed with the FCS Express software (DeNovo Software). Representative density plots were normalized on equal numbers of events occurring in the gates of interest. 3D bioprinted scaffold without cells was stained with the antibodies we used, in order to exclude nonspecific binding.

### Immunohistochemistry and Fluorescent Images

3D bioprinted scaffolds containing CLL primary cells after 7 days of culture were washed twice with HBSS with 50mM of CaCl_2_ for 8 minutes at 37°C, and then fixed with 4% PFA containing 50mM of CaCl_2_ o/n at 4C. After fixation, scaffolds were washed twice with HBSS with 50mM of CaCl_2_ for 10 minutes RT and eventually incubated, first, for 45 minutes at 4°C in HBSS with 50mM, then for another 45 minutes RT in sucrose 30% in PBS. The scaffolds were then embedded in OCT matrix and placed at -80°C until cryosectioning. Frozen samples were sectioned (5-7µm) on Superfrost-plus microscope slides (Thermo Fisher Scientific, Massachusetts, USA), washed one time with PBS, and then stained with Mayer’s Hematoxylin (Bio-Optica, Milan, Italy) for 1 minute, washed with tap water and stained with Eosin G (Bio-Optica, Milan, Italy) for 2 minutes. Images were taken with Zeiss Axio Imager M2m microscope with AxioVision (Rel. 4.9.1) software, and then processed using FIJI (ImageJ) software. Images of MEC-GFP cells inside and outside the 3D bioprinted scaffolds were obtained by using JuLI™ Stage fluorescent microscope.

### Statistical Analysis

Student’s t-test was performed for statistical analysis (GraphPad Prism v.8.0a). Mann-Whitney unpaired t test was used for non-parametric comparisons of data sets (*p < 0.05; **p < 0.01; *** p < 0.001; ****p < 0.0001).

## Results

### 3D Bioprinting Supports CLL Cells Viability

We initially used the CLL cell line MEC1 (21) to set up the 3D bioprinting strategy and the analytic protocols, considering that there were no previous studies showing the printability of lymphocytes and their behavior in these settings of 3D culture.

**Figure 1:**
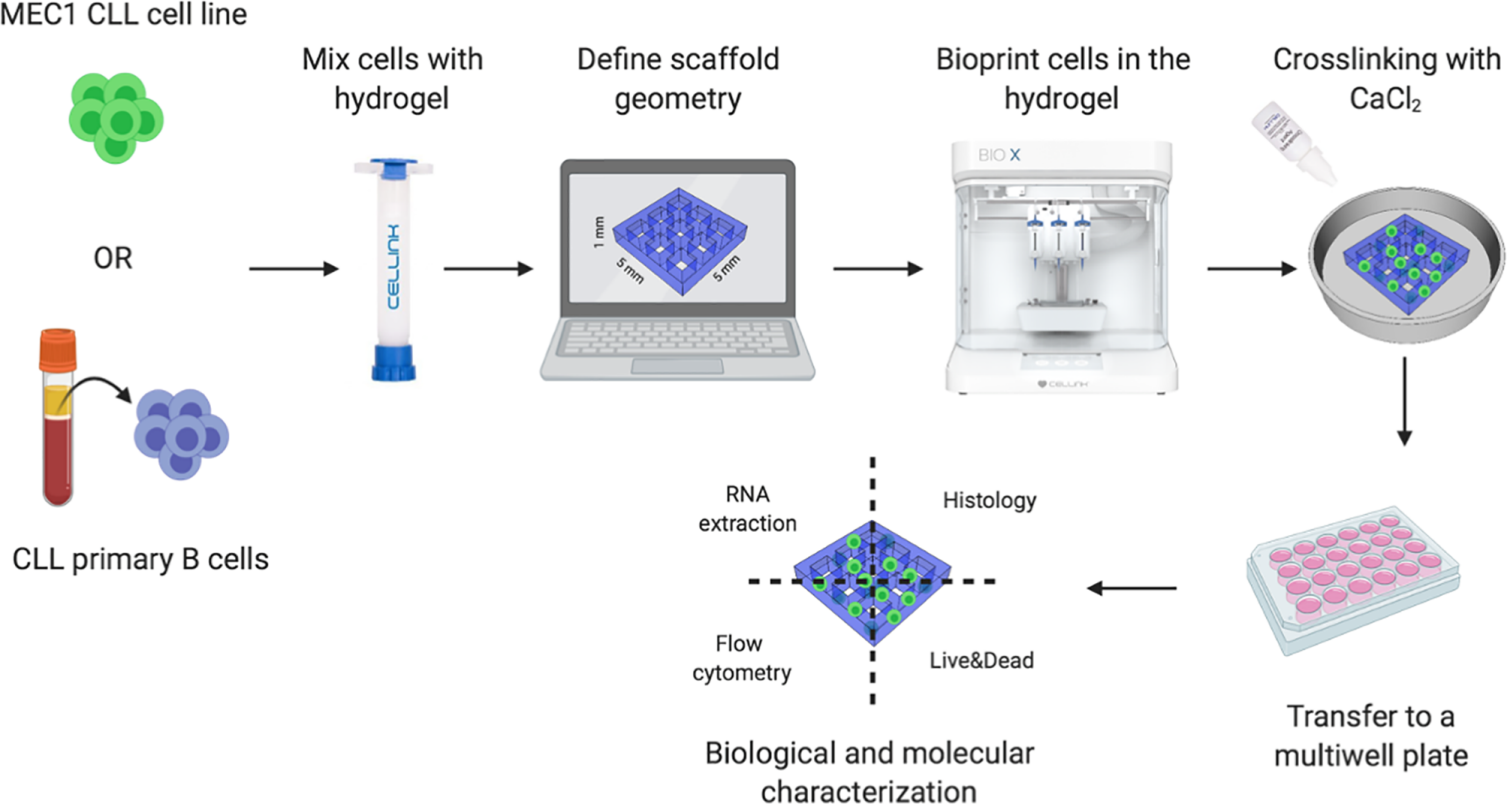
3D bioprinting strategy. Schematic representation of our 3D bioprinting strategy: CLL cell line MEC1 or CLL primary B cells were used. The cells are pre-mixed with hydrogels specifically designed to support cellular adhesion and functions, as well as high printability and biocompatibility. Once the geometry of the 3D bioprinted scaffolds is defined, the cells are printed, encapsulated in the hydrogel matrix, crosslinked with CaCl_2_ and placed in a culture plate. The constructs are then processed to extract the RNA, perform histological analyses, assess cell surface markers and cell viability.

**Figure 2:**
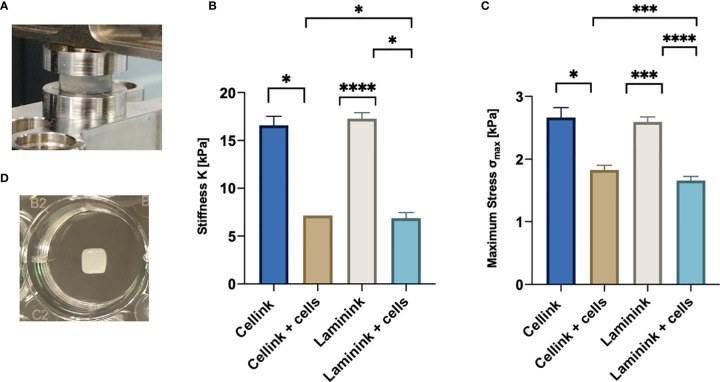
Compressive mechanical properties of hydrogel scaffolds with and without embedded cells. **(A)** Hydrogel scaffold specimen in the compression mode clamps. **(B)** Average and standard deviation values of stiffness, K, for the scaffolds (n=4). **(C)** Average and standard deviation values of maximum stress, σ_max_ (n=4). **(D)** Representative picture of a 5x5x1mm^3^ 3D-bioprinted scaffold in a well of a 24-well plate. *p < 0.05, ***p < 0.001, ****p < 0.0001.

First of all, we had to define the optimal number of lymphoid cells to be 3D bioprinted proportionally to the hydrogel quantity. Following a theoretical calculation (see *Materials and Methods* section for details) and based on cells dilution experiments (data not shown), we defined that the ratio of 10 x 10^6^ MEC1 cells per 100µl of hydrogel was the optimal cell density, to adequately fill the scaffold but also to leave enough space for subsequent cell proliferation and eventually for the deposition of the extracellular matrix. To print MEC1 cells, we used CELLINK Bioink hydrogel, which is specifically designed to support cellular adhesion and functions, as it has high printability and biocompatibility (**Supplementary Table 2**). The cells were premixed with the hydrogel; we designed the geometry of the 3D bioprinted scaffold with Fusion 360 software and, eventually, we bioprinted the cells encapsulated in the hydrogel matrix. The pressure applied to the 3D bioprinting process is hydrogel/cells-dependent and a range of values around 11-14 kPa was used for our setting (see *Materials and Methods* section). The resulting scaffold was then cross-linked with CaCl_2_ in order to give the needed stiffness and it was placed in a traditional culture plate (**Figure 1**). We measured the stiffness of the CELLINK Bioink hydrogel with and without cells (n = 4); stiffness values were found ranging around 16 and 7 kPa for cell-free and cellularized scaffolds, respectively, and lower maximum stress at 30% strain (**Figure 2**), showing a significant difference comparing 3D printed scaffold without cells and after 7 days of culture. In particular, the stiffness values matched the expected ones for the lymphoid tissues of our interests (26).

**Figure 3:**
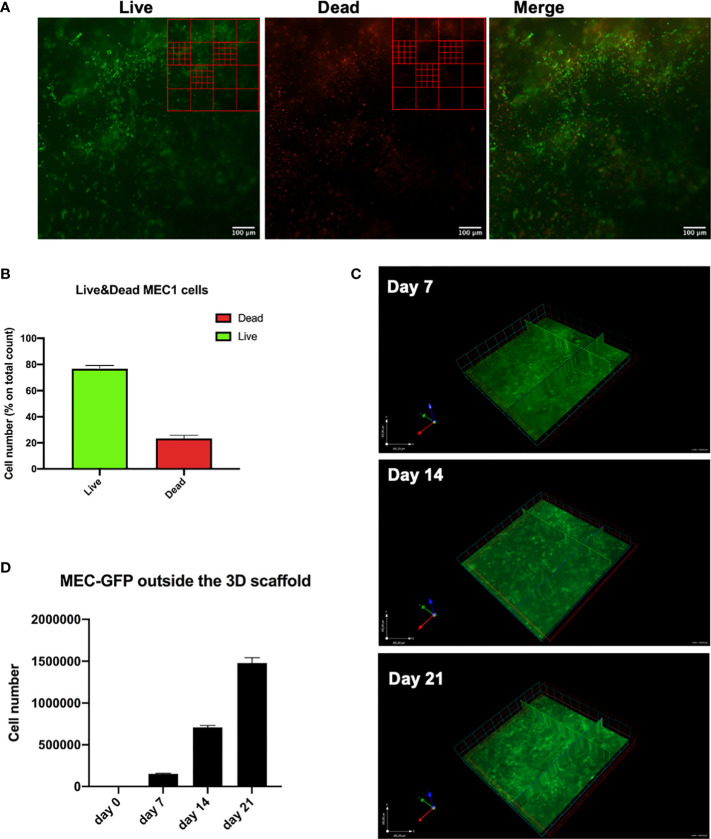
CLL cells are viable and homogeneously distributed in the hydrogel matrix. MEC1 and/or MEC-GFP cell lines were used to define the optimal number of cells and hydrogel quantity to be printed. **(A)** Representative images of Live/Dead assay of 3D bioprinted MEC1 cell line acquired with Axio Observer Zeiss fluorescent microscope. Green cells are alive cells; red cells are dead cells. An example of a grid scheme used to quantify alive and dead cells is shown on the top right of each image. **(B)** The graph shows quantification of alive (green column portion) and dead (red column portion) 3D bioprinted MEC1 cells. **(C)** Representative z-stack images of 3D bioprinted MEC-GFP cells in the scaffold overtime showing their distribution. Images were obtained with Axio Observer Zeiss fluorescent microscope. **(D)** The graph shows quantification by cell count, at different time points (day 0-7-14-21), of viable MEC-GFP cells found outside the 3D bioprinted scaffold. Data are represented as mean ± SEM, n=3 **(B)** and n=5 **(D)**.

Soon after printing (after 7 days), we performed a Live/Dead assay that allowed us to discriminate viable and dead MEC1 cells quantified by drawing a grid on the images acquired by fluorescent microscope, and counting the cells manually (**Figure 3A**). About 75% of the cells in the printed scaffolds were alive (**Figure 3B**), thus demonstrating that CLL cells can be efficiently 3D bioprinted and that the printing process has only limited effects on their viability.

In order to visualize the spatial organization of the cells inside the 3D bioprinted hydrogel matrix, we used GFP-labelled MEC1 cell line (MEC-GFP) (22): we detected MEC-GFP cells homogeneously distributed throughout the 3D structure (**Figure 3C**).

Furthermore, we tested the ability of 3D bioprinted cells (MEC-GFP cells) (n=5) to move throughout the hydrogel scaffold after the print, up to 3 weeks of culture. Specifically, starting from day 0 to day 21 after printing, MEC-GFP were still found inside the scaffold (**Figure 3C**) but also outside, floating in the medium (0; 1,5x10^5^; 7x10^5^; 14,8x10^5^ cells at day 0, 7, 14, 21, respectively) (**Figure 3D**). MEC-GFP cells might be found outside the scaffold, by actively moving throughout the hydrogel as a consequence of the expansion due to their proliferation or by passively diffusing outside the hydrogel matrices because of its spontaneous degradation after a few days in culture (27–30).

Moreover, once MEC-GFP cells leave the scaffold, they maintain the characteristic phenotype of the cell line as demonstrated by their typical growth in clusters when cultured in suspension (**Supplementary Figure 2A**).

### 3D Bioprinted and 2D Cultured MEC1 Cells Show Differences in Gene Expression

**Figure 4:**
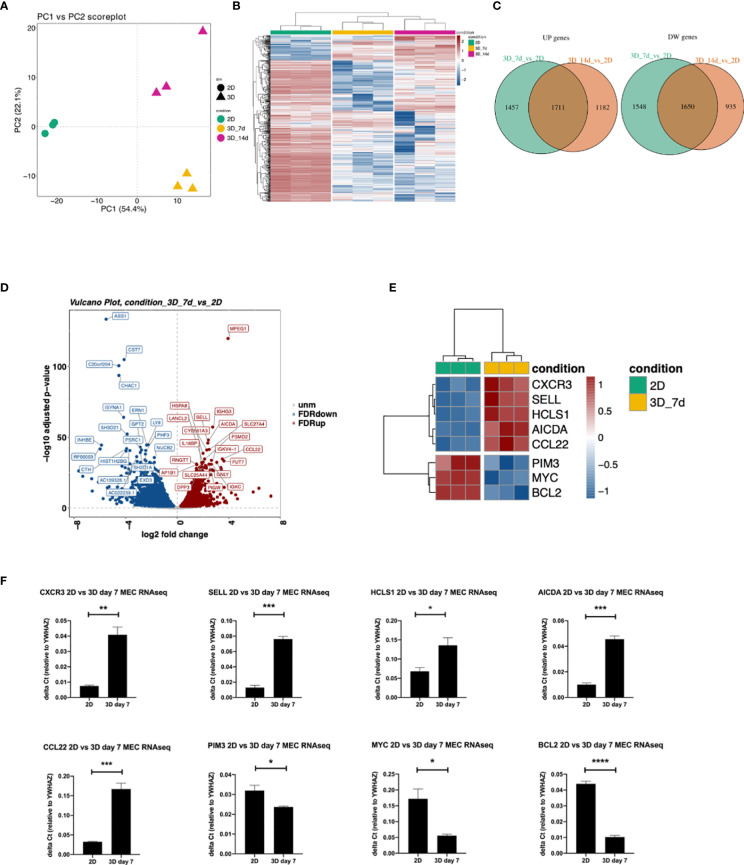
Differences in gene expression between 3D bioprinted and 2D cultured MEC1 cells: clustering analysis and top genes validation. **(A)** PCA plot built using the 500 most variable genes (in RPKM). **(B)** Heatmap of the 500 most variable genes (in RPKM), clustering row (genes) and columns (samples). Expression is scaled. **(C)** Intersection between up-regulated (left) and down-regulated (right) genes between the comparisons 3D_7d_vs_2D and 3D_14d_vs_2D. **(D)** Vulcano plot for the comparison 3D_7d_vs_2D highlighting the 20 most significantly up-regulated (red) and down-regulated (blue) features for the FDR filter. **(E)** Heatmap summarizing genes among the first 100 most modulated in the comparison 3D_7d_vs_2D. **(F)** The graphs show mRNA levels of n=8 genes selected among the first 100 most modulated in the comparison 3D_7d_vs_2D which have been validated by RT-qPCR (*AICDA, SELL, CXCR3, CCL22, HCLS1, PIM3, MYC, BCL2*). *p < 0.05, **p < 0.01, ***p < 0.001, ****p < 0.0001. Data are represented as mean ± SEM, n=3 MEC1 cell line samples. Student’s t-test was performed for statistical analysis.

We performed RNAseq analysis to compare 3D bioprinted to 2D cultured MEC1 cells, in order to evaluate genes and pathways affected by the 3D printing strategy. The Principal Component Analysis (PCA) was performed, and the two principal components were identified (PC1 and PC2) by using the 500 most variable genes in terms of RPKM (reads per kilobase of transcript per million reads mapped) (31). Specifically, we observed a clear segregation of the samples according to the different conditions: 2D and 3D samples substantially separated along PC1, expressing 54,4% of the total variance, while the effect of the considered 3D time points (day 7 vs day 14) is evident along PC2, representing 22,1% of the total variance (**Figure 4A**). Similarly, we observed a visible different effect of the analyzed conditions in the heatmap (**Figure 4B**), showing a strong separation between 2D and 3D bioprinted samples in terms of gene expression.

By performing the differential expression analysis with the package DESeq2 (24) using FDR (False Discovery Rate) as the cut-off to determine the significance of the differential genes (32), we detected a high number of modulated genes in the 2D vs 3D conditions, some up-regulated and some down-regulated (**Figure 4C**). Among the first 100 most modulated genes, we identified genes with particular interest for CLL pathophysiology (**Figures 4D, E****)**, which we successfully validated by RT-qPCR (**Figure 4F**). In detail, we observed upregulation in 3D culture of the following genes: *CXCR3*, chemokine receptor that is involved in cellular responses, leukocyte trafficking, integrin activation, cytoskeletal remodeling (33), and its expression has been demonstrated to play a prognostic role in CLL (34); *CCL22*, cytokine that has been demonstrated to be produced by CLL cells to chemo attract T lymphocytes (35, 36); *SELL*, gene that encodes for a Calcium-dependent lectin that mediates the adherence of lymphocytes to endothelial cells in peripheral lymph nodes and promotes the initial tethering and the rolling of leukocytes in endothelium and has been recently demonstrated to be involved in CLL transformation to high-grade B-cell lymphoma (37, 38); *HCLS1*, gene that we demonstrated in the past being involved in cytoskeletal remodeling, migration, trafficking and homing of CLL cells (16, 22); *AICDA*, gene that is involved in somatic hypermutation, gene conversion, and class-switch recombination in B-lymphocytes, and it is required for several crucial steps of B-cell terminal differentiation necessary for efficient antibody responses (39, 40). Interestingly, we also observed downregulation of: *MYC*, proto-oncogene that plays a central role in cell cycle progression, apoptosis and cellular transformation and promotes VEGFA production and subsequent sprouting angiogenesis, its role has been recognized in the transformation in aggressiveness of indolent B cell malignancies (41, 42); *PIM3*, proto-oncogene overexpressed in hematological and epithelial tumors and associated with MYC, their coexpression has a role in the regulation of signal transduction cascades, contributing to both cell proliferation and survival, and provides a selective advantage in tumorigenesis (43). The last gene that we validated for its importance in CLL is *BCL2* gene that is downregulated in 3D cultured MEC1 cells and encodes for a protein that suppresses apoptosis in a variety of cellular systems including factor-dependent lymphohematopoietic and neural cells, thus it is used as a therapeutic target for B-cells malignancies in particular in CLL (44).

**Figure 5:**
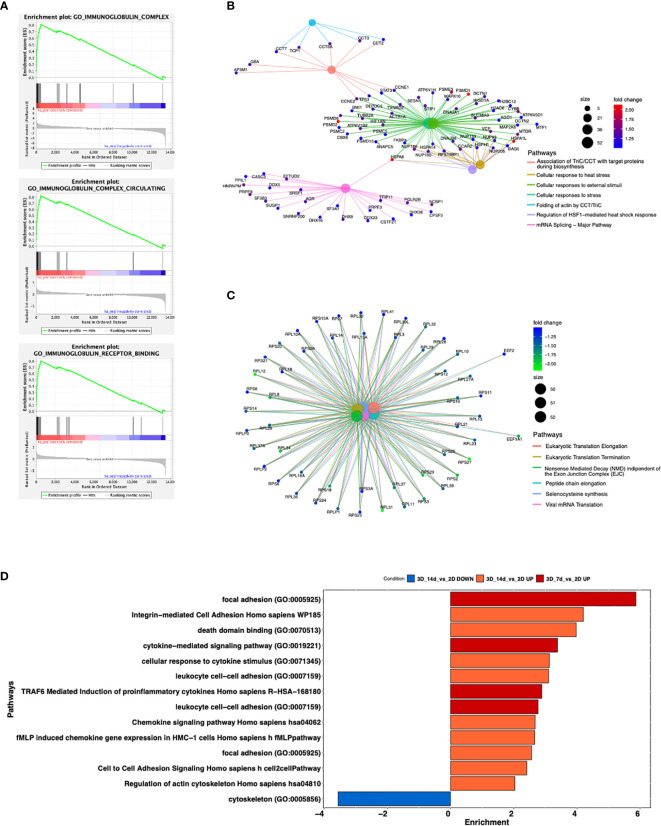
Enrichment analysis of significantly modulated genes enriching different pathways. **(A)** GSEA curves for the first most significantly up-regulated pathways for Gene Ontology database in the comparison 3D_7d_vs_2D. **(B, C)** Cnetplots highlighting up-regulated **(B)** and down-regulated **(C)** pathways between the comparisons 3D_7d_vs_2D. **(D)** Bar plot showing most modulated pathways in both comparisons 3D_7d_vs_2D and 3D_14d_vs_2D.

By Gene Ontology analysis, we further investigated pathways potentially affected by the different culture conditions. We observed that 3D cultured cells at both day 7 and 14 show increased production of immunoglobulins, a key feature for CLL pathobiology and current therapeutic target (45–47) (**Figure 5A** and **Supplementary Figure 3A**) pro-inflammatory molecules (e.g. IFN alpha and beta), immune response activation and cellular stress markers with respect to 2D ones (**Figure 5B** and **Supplementary Figure 3B**), paralleled by a decreased production of proteins involved in protein targeting the Endoplasmic Reticulum (ER) and cell membrane, DNA replication, organelle fission, protein translation and cell division respect to 2D ones (**Figure 5C** and **Supplementary Figure 3C**). Interestingly, we found upregulated genes enriching for pathways involved in the formation of focal adhesion, as well as in the production and activation of cytokines/chemokines, and cell-cell adhesion at both day 7 (**Figure 5D** and **Supplementary Figure 3D****)** and day 14 in 3D compared to 2D (**Figure 5D** and **Supplementary Figure 4**), suggesting a strong adaptation of the cell line MEC1 in the 3D culture.

### 3D Bioprinted Primary CLL Cells Show a Long-Term Viability

Once we set up the 3D bioprinting strategy for the MEC1 cell line, we transitioned to primary B lymphocytes isolated from the peripheral blood (PB) of patients affected by CLL and healthy donors.

First, we evaluated primary CLL cells printability by testing different hydrogels (CELLINK AB) that could favor CLL cells survival, namely: RGD10, Lam111, Lam411 and Lam521; we embedded 20x10^6^ primary CLL cells in 100µl of hydrogel. The Alamar blue viability assay showed that leukemic cells may survive for up to 7 days in all considered matrices (**Supplementary Figures 5A–D**), showing higher and thus more promising viability values [expressed in Fluorescence mean value (FM)] in the hydrogel matrix containing laminin 411, after 7 days of culture (FM Lam411 = 876103 vs FMs RGD10 = 388774, Lam111 = 492252, Lam521 = 394100) (**Supplementary Figure 5E**). Taken together, these results led us to conclude that CELLINK Laminink411 hydrogel was the most suitable for our study on primary CLL cells. Indeed, we also tested MEC1 cells printability in the laminin hydrogel series but we didn’t observe an improvement in cell survival in comparison with CELLINK Bioink (data not shown).

Stiffness of CELLINK Laminink411 hydrogel was found in the range of 7 and 17 kPa for scaffolds printed with and without cells, respectively (**Figure 2**). Values measured on cellularized scaffolds were similar to what expected in the lymphoid tissues of our interests (19).

To study a larger cohort of patients (n = 26), we selected them based on the mutational status of the IGHV gene (IGHV<98%=mutated=good prognosis (mCLL) n = 16; IGHV≥98%=unmutated=bad prognosis (uCLL) n = 9) (48), in order to evaluate possible differences in the outcomes of their PB-derived cells behavior *in vitro* (**Supplementary Table 1**).

**Figure 6:**
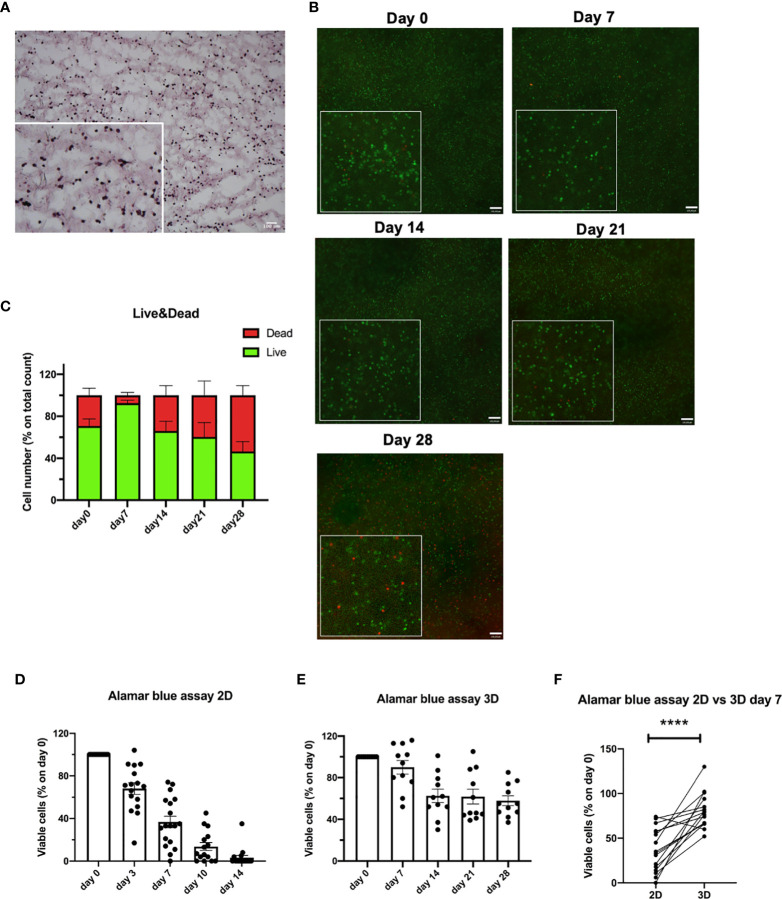
3D bioprinted CLL primary cells show a long-term viability in the scaffolds. **(A)** Representative H&E staining on 5 µm frozen sections of 3D bioprinted CLL primary cells showing their distribution in the scaffold. Images were obtained with Zeiss Axio Imager M2m microscope. A magnification of the image is shown. **(B)** Representative images of Live/Dead assay of 3D bioprinted CLL primary cells at different time points (day 0-7-14-21-28) acquired with Axio Observer Zeiss fluorescent microscope. Green cells are live cells; red cells are dead cells. A magnification for each image is shown. **(C)** The graph shows quantification, at different time points (day 0-7-14-21-28), of alive (green column portion) and dead (red column portion) 3D bioprinted CLL primary cells. **(D)** The graph shows Alamar blue assay fluorescence values of 2D cultured CLL primary cells at different time points (day 0-3-7-10-14). **(E)** The graph shows Alamar blue assay fluorescence values of 3D bioprinted CLL primary cells at different time points (day 0-7-14-21-28). **(F)** The graph shows the percentages of viable 3D bioprinted CLL primary cells compared to CLL primary cells cultured in the traditional 2D system. Cell viability was measured by Alamar blue assay after 7 days of culture and normalized to day 0. ****p < 0.0001. Data are represented as mean ± SEM, n=16 **(D, F)** and n = 11 **(E)** patient samples. Paired t-test was performed for statistical analysis.

Primary CLL cells were 3D bioprinted in the CELLINK Laminink411 hydrogel, placed in standard culture conditions without the addition of any stimuli, and their viability was evaluated over time (up to 28 days). First, we confirmed a homogeneous distribution of 3D bioprinted primary CLL cells by performing H&E staining on a 5µm frozen section (**Figure 6A**). Then, by performing Live/Dead assay, we observed viability up to 28 days for 3D bioprinted primary CLL cells (n = 8), independently of their clinical/biological features (**Figure 6B**). In detail, using the Live/Dead assay, we observed 93%, 66%, 69%, 47% of viable cells at 7, 14, 21, 28 days after the print, respectively (**Figure 6C**). This data was confirmed by Alamar blue viability assay (n = 11) that showed 90%, 63%, 62%, 58% of live cells at the same Live/Dead assay time points (**Figure 6E**).

Notably, we also performed a Live/Dead assay on the scaffolds (n = 3 patients) that we cut in half and then images were acquired at all the time points (0-7-14-21-28 days), and we observed that cells are viable in the entire scaffold (**Supplementary Figure 6**).

In contrast, when we evaluated the viability over time (up to 14 days) of traditionally 2D cultured primary CLL cells (n = 16), grown on Laminin411 coated tissue culture plates without the addition of exogenous factors, we observed a dramatic decrease in cells viability after a few days in culture: 68%, 37%, 14%, 4% of viable cells by Alamar blue assay at 3, 7, 10, 14 days after the print, respectively (**Figure 6D**).

When we compared 2D cultured with 3D bioprinted primary CLL cells viability after 7 days of culture (n = 16), a time point when a proportion of 2D cultured cells is still viable, 3D bioprinted primary CLL cells showed a significantly higher viability by Alamar blue assay compared to 2D cultured ones (p < 0.0001) (**Figure 6F**). Interestingly, primary CLL cells derived from mCLL were found to be significantly more viable (p = 0.0006) than those obtained from uCLL (p = 0.0115) (**Supplementary Figures 7A, B****)**.

In parallel, we tested the ability of 3D bioprinted CLL cells (n = 3) to move throughout the hydrogel scaffold after the print, up to 4 weeks of culture. We noted that CLL cells were virtually absent outside the scaffold, thus supporting the hypothesis that matrix degradation is not the main cause for the presence of cells in the supernatant, which can be explained by active movement through the matrix in response to the increased number of proliferating cells that is not taking place in the case of primary resting CLL cells.

Notably, we tested the possibility of 3D bioprint Peripheral Blood Mononuclear Cells (PBMCs) from healthy donors in the Cellink Laminink411 hydrogel, and we observed also in this case a sustained viability over time (up to 28 days) (**Supplementary Figure 8**), thus paving the way to the use of this approach in the study of both healthy and malignant lymphocytes.

### The CLL Cells Phenotype Is Preserved Throughout the 3D Culture

**Figure 7:**
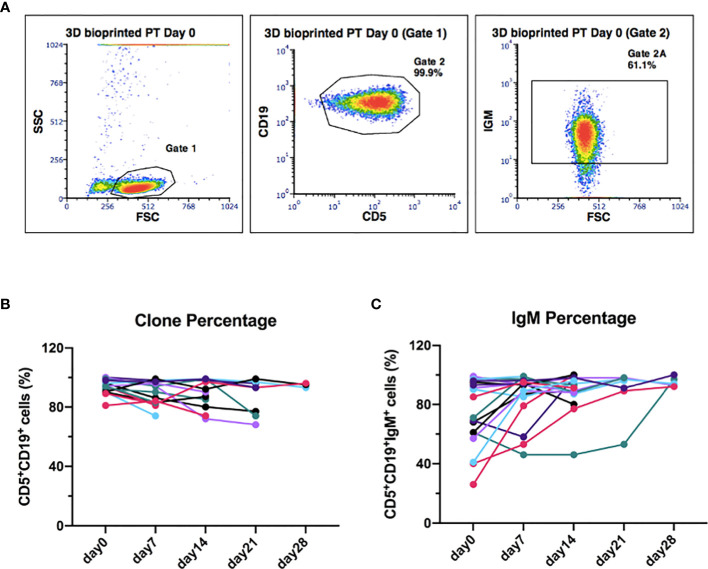
The original CLL clone phenotype CD19/CD5 is maintained throughout the 3D culture, including the levels of IgM. **(A)** Representative flow cytometry plots of 3D-bioprinted CLL primary cells (day 0) showing the presence of the leukemic clone and the surface marker IgM, based on the expression of CD19/CD5 and IgM surface markers. Physical parameters of 3D bioprinted hydrogel alone are shown as well. **(B)** The graph shows the leukemic clone percentage of all samples analyzed overtime (n = 15). **(C)** The graph shows IgM surface marker percentage of all samples analyzed overtime (n = 18).

To further validate our 3D model of leukemic B cells, we evaluated, by flow cytometry, the ability of 3D bioprinted primary CLL cells to maintain the characteristic surface phenotype throughout the whole culture period. We observed that the CLL clone maintained CD19 and CD5 expression during the whole culture period, with mean values of 93%, 89%, 89%, 87%, 95% for day 0-7-14-21-28, respectively (**Figures 7A, B** and **Supplementary Figures 9A, B****)** (n = 15). The same observation can be done for surface IgM expression (**Figures 7A, C** and **Supplementary Figures 9A, C****)** (n = 18), whose levels showed a trend toward an increase in 15 patients (7 uCLL and 8 mCLL) out of 18 cases analyzed (10 uCLL and 8 mCLL). The increment observed on the percentage of IgM+ cells was about 10-20% from day 0 to the following time points visualized in **Figure 7C** (mean % of IgM+ cells weekly increase from day 0 to day 28, respectively: 34%, 16%, 5%, 4%). We didn’t observe any statistical differences between mutated and unmutated cases.

### 3D Bioprinted Primary CLL Cells Show *BAX* and *BCL2* Regulation in Culture

**Figure 8:**
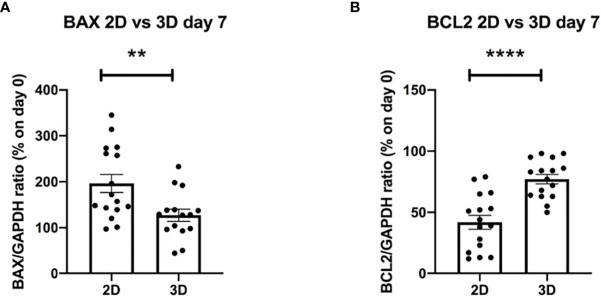
The evaluation of apoptotic genes shows better survival of 3D bioprinted primary cells compared to 2D cultured ones at the same time of culture. **(A, B)** The graphs show mRNA levels of pro-apoptotic gene *BAX* and anti-apoptotic gene *BCL2*, after 7 days of culture, of 3D bioprinted CLL primary cells compared to CLL primary cells cultured in the traditional 2D system. **p < 0.01, ****p < 0.0001. Data are represented as mean ± SEM, n = 16 patient samples. Mann-Whitney t-test was used for statistical analysis.

To further elucidate the biological mechanisms at the basis of the improved viability shown in the 3D system, we decided to evaluate possible changes in the levels of expression of genes known to be involved in the apoptotic process, such as *BAX* and *BCL2* (49) (**Supplementary Figures 10A, B**) (n = 10). Interestingly, we observed significantly lower values of the pro-apoptotic gene *BAX* and significantly higher values of the anti-apoptotic gene *BCL2* in 3D bioprinted CLL primary cells as compared to 2D cultured ones, analyzed after 7 days, independently of their clinical/biological features (**Figures 8A, B****)** (n = 16).

## Discussion

In the present work, we tested for the first time whether 3D bioprinting strategy could be applied to immune cells and in particular to leukemic B cells.

This strategy is currently successfully explored for other cancers such as breast, brain, skin and pancreatic (18). In solid tumors, researching the need to develop *in vitro* models with a 3D structure recapitulating *in vivo* tumor growth was more obvious, while this need was appreciated much later in hematological cancer, due to the circulating nature of most diseases (2, 3). It is now evident that leukemia cells in the peripheral blood do not represent entirely the disease, that is composed by the cells accumulating and proliferating in lymphoid tissues (4).

Several 3D systems have been recently applied to the study of different B cell malignancies in order to recapitulate the tissue environment, including spheroids (50), organoids and microfluidic devices (51). In particular, the BM microenvironment niche has been more frequently investigated as in the case of acute myeloid leukemia and multiple myeloma in order to study the resistance to chemotherapeutics. For CLL rather limited attempts have been made by using co-culture with stromal cells in both spheroids (52) or gelatin scaffolds, the latter kept in dynamic growth in bioreactor (16).

However, all these systems have their intrinsic limitations such as the need of cell seeding. Indeed, the main difference between 3D printing and other 3D culture systems is that multiple cell types can be deposited with a microscale precision (53). We believe that before exploring the possibility to print different cell types in the hydrogel to generate complex 3D scaffolds to study CLL, we need to understand how CLL cells behave in this context.

To this aim we directly printed CLL cells embedded in hydrogels specifically designed to support cellular adhesion and functions, as well as high printability and biocompatibility. We successfully tested CLL cells for printability, optimized the printing strategy and set-up the protocols to perform the analysis (cell viability assays, protein/gene expression, imaging among others). The CLL cell line MEC1 was used to define the optimal number of cells and cell/hydrogel ratio to be printed, followed by validation of the derived settings using primary cells obtained from the PB of patients affected by CLL, which we selected based on the mutational status of the IGHV gene (IGHV<98%=mutated=good prognosis (mCLL); IGHV≥98%=unmutated=bad prognosis (uCLL); **Supplementary Table 2**).

We observed a different printability of the cell line with respect to primary cells: in particular, MEC1 cells showed better behavior in the hydrogel without the addition of external factors (Laminins), while CLL cells prefer hydrogels with the addition of laminins. This result suggests and confirms that primary cells are still highly dependent on the microenvironment, including extracellular matrix (7).

In general, CLL cells, both cell line and primary cells, were found to be homogeneously distributed in the hydrogel scaffolds and, specifically, we observed a long-term viability (up to 28 days) for primary CLL cells cultured in the 3D bioprinted hydrogel, independently on their clinical/biological features, result that is not achievable when CLL cells are cultured in 2D alone.

Of note, CLL cells appear to be indeed affected by the 3D culture in the presence of a sort of extracellular matrix, as indicated by the strikingly different expression profile shown by RNAseq analysis when we compared cells cultured in 2D and 3D cultures. In particular, the upregulation of Immunoglobulin complexes (45–47), the activation of integrins (54), inflammation and cytoskeletal (55) related pathways suggest that the cells possibly lay in a more physiological environment. It is clear that the traditional 2D culture system does not represent the proper control for our 3D model, and it will be interesting to see how leukemic cells mirror e.g. cells obtained *ex vivo* from BM or LN from patients in terms of expression profile and functional behavior, and to understand which pathways might be more affected than others in the presence of extracellular matrix alone.

To this aim, we measured the stiffness of the hydrogel used in our experiments as we believe that this will be a fundamental parameter to be studied in the tissues of origin, since it could influence the maintenance/promotion of CLL cells viability. In the future, this could lead to the development of smart materials with tuned stiffness to be used for 3D bioprinting cells of different origin(s).

Focusing on primary CLL cells, we observed that the well-being of the 3D bioprinted cells is also suggested by their phenotype (expression of surface CD19 and CD5) that remains unaffected through the culture period. Inspired by the RNAseq results, we measured the levels of the B cell receptor IgM that increases in 15/18 patients analyzed. This is intriguing considering that Coulter et al. (56) recently reported that the B cell receptors of LN and PB derived CLL cells might be functionally distinct, in particular LN-CLL cells express higher levels of surface IgM thus suggesting that the 3D environment may more reliably reproduce this particular *in vivo* environment. Similarly, cells showed higher levels of *BCL2* expression in 3D vs 2D after 7 days of culture, with lower levels of *BAX* indicating a higher viability and less priming toward apoptosis. The overall higher fitness of the cells is crucial, especially when considering the potential application of this technique to study the response of CLL cells *ex vivo* to different drugs and immunotherapeutic approaches. Keeping in mind that *BCL2* is a therapeutic target in CLL (57), this system might potentially be more reliable in predicting responses in patients and also to understand how cells may adapt to the presence of the drug with time, when cultured in a more protective microenvironment.

In summary, our results demonstrate that we can efficiently 3D bioprint primary CLL cells and healthy lymphocytes, and improve their viability, which can be maintained for up to 28 days, thus establishing the first long-term 3D culture model for leukemia cells. Considering that no similar approach has been so far established also for normal B lymphocytes, this is an innovative tool that may help better mimic the physiological *in vivo* settings not only of leukemic lymphocytes but also of immune cells in general. In the future, the system can be further improved by increasing the complexity of the cellular and molecular components to be included in the 3D bioprinted models in order to even better recapitulate a tumor microenvironment. This could allow to recreate the molecular and cellular interactions that occur in normal and neoplastic conditions *in vivo*, and most importantly could be exploited to test individual response(s) to different drugs such as target therapies or immunotherapy.

## Data Availability Statement

The RNAseq data, including raw sequence files, have been submitted to NCBI’s Gene Expression Omnibus and are accessible through the GEO series accession number GSE163977.

## Ethics Statement

The studies involving human participants were reviewed and approved by the Ospedale San Raffaele (OSR) ethics committee under the protocol VIVI-CLL entitled: “In vivo and *in vitro* characterization on CLL.” The patients/participants provided their written informed consent to participate in this study.

## Author Contributions

CS, FVS, and RP wrote the manuscript. CS, FVS, RP, FB, DR, and SF performed the experiments and analyzed the data. LS and PG provided patients’ and clinical information. FS, INR, HM, SF, and PG revised the manuscript. All authors contributed to the article and approved the submitted version.

## Conflict of Interest

Authors INR and HM were employed by CELLINK AB.

The remaining authors declare that the research was conducted in the absence of any commercial or financial relationships that could be construed as a potential conflict of interest.
