# Supplementary material for: 3D Bioprinting Allows the Establishment of Long-Term 3D Culture Model for Chronic Lymphocytic Leukemia Cells
Source: Front Immunol. 2021 May 3;12:639572. doi: 10.3389/fimmu.2021.639572 (PMC8126722; doi:10.3389/fimmu.2021.639572)
Supplement: Supplementary file 1 [file DataSheet_1.pdf]

3D Bioprinting allows the establishment of long-term 3D culture model for Chronic Lymphocytic Leukemia cells

Francesca Vittoria Sbrana[1], Riccardo Pinos[1,2], Federica Barbaglio[1], Davide Ribezzi[1,3], Fiorella Scagnoli[1], Lydia Scarfò[2,4], Itedale Namro Redwan[5], Hector Martinez[5], Silvia Farè[3], Paolo Ghia[2,4] and Cristina Scielzo[1]

Supplementary information

Supplementary table 1

Patient n°	Provenience	Sample	Application	Rai	Binet	CD38 result	IGHV identity	FISH	Clinical course
28	OSR	PB	Viability assays, qRT-PCR, FC	0	A	82,0	89,24	del(13q)	PROGRESSIVE
51	OSR	PB	Viability assays, qRT-PCR	0	A	2,1	97,00	del(13q), del(11q)	PROGRESSIVE
82	OSR	PB	Viability assays, qRT-PCR, FC	1	A	97,0	97,22	del17p (8%), del13q34 (7.9%)	STABLE
123	OSR	PB	Viability assays, qRT-PCR, FC	0	A	0,2	92,71	del(13q)	STABLE
138	OSR	PB	Viability assays, qRT-PCR, FC	0	A	3,0	88,90	N. A.	PROGRESSIVE
188	OSR	PB	Viability assays, qRT-PCR, FC	0	A	0,6	96,0	del(13q)	STABLE
199	OSR	PB	Viability assays, qRT-PCR	0	A	16,3	100,00	del(17p)	PROGRESSIVE
211	OSR	PB	Viability assays, qRT-PCR, FC	0	A	0,4	100,00	del(13q)	STABLE
214	OSR	PB	Viability assays, qRT-PCR, FC	0	A	24,8	100,00	del(13q)	PROGRESSIVE
219	OSR	PB	Viability assays, qRT-PCR, FC	0	A	1,2	100,00	Normal	PROGRESSIVE
225	OSR	PB	Viability assays, qRT-PCR, FC	0	A	15,9	98,00	Normal	PROGRESSIVE
236	OSR	PB	Viability assays, qRT-PCR	0	A	0,0	96,26	del(13q)	STABLE
317	OSR	PB	Viability assays	0	A	0,3	95,79	N. A.	STABLE
324	OSR	PB	Viability assays	0	A	0,2	92,01	N. A.	STABLE
358	OSR	PB	Viability assays, qRT-PCR, FC	1	N. A.	0,0	91,30	del(13q)	PROGRESSIVE
359	OSR	PB	Viability assays, qRT-PCR	0	A	17,8	99,70	N. A.	STABLE
370	OSR	PB	qRT-PCR, FC	0	A	2,3	100,00	del(13q)	PROGRESSIVE
376	OSR	PB	Viability assays, qRT-PCR, FC	1	A	15,5	100,00	del(17p)	PROGRESSIVE
440	OSR	PB	Viability assays, qRT-PCR, FC	0	A	0,2	96,49	N. A.	STABLE
485	OSR	PB	Viability assays, qRT-PCR, FC	1	B	5,9	96,22	del(13q)	PROGRESSIVE
498	OSR	PB	Viability assays	0	A	37,5	97,57	N. A.	STABLE
511	OSR	PB	Viability assays, qRT-PCR, FC	0	A	74,9	100,00	Normal	PROGRESSIVE
579	OSR	PB	Viability assays	1	B	67,3	96,18	N. A.	STABLE
575	OSR	PB	Viability assays	N. A.	N. A.	N. A.	97,94	N. A.	STABLE
595	OSR	PB	Viability assays	N. A.	N. A.	N. A.	N. A.	N. A.	STABLE
616	OSR	PB	Viability assays	N. A.	N. A.	N. A.	96,2	N. A.	STABLE

Supplementary table 1. Clinical and biological characteristics of the patients used for the experiments presented in the manuscript (n=26). N.A. = Not Available

Supplementary table 2

	Composition	Printing temperature	Crosslinking	Crosslinking time (minutes)
**CELLINK Bioink**	Sodium alginate + Nanofibrillar cellulose + D-mannitol + HEPES buffer solution	RT	50mM $CaCl_2$	4
**CELLINK RGD10**	Sodium alginate + Nanofibrillar cellulose + D-mannitol + HEPES buffer solution + synthetic peptides	RT	50mM $CaCl_2$	4
**CELLINK Laminink111**	Sodium alginate + Nanofibrillar cellulose + Laminin 111 + D-mannitol + HEPES buffer solution	RT	50mM $CaCl_2$	4
**CELLINK Laminink411**	Sodium alginate + Nanofibrillar cellulose + Laminin 411 + D-mannitol + HEPES buffer solution	RT	50mM $CaCl_2$	4
**CELLINK Laminink521**	Sodium alginate + Nanofibrillar cellulose + Laminin 521 + D-mannitol + HEPES buffer solution	RT	50mM $CaCl_2$	4

Supplementary table 2. List of utilized hydrogels, their composition, printing temperature, crosslinker agent and time of crosslinking.

Supplementary figure 1

A

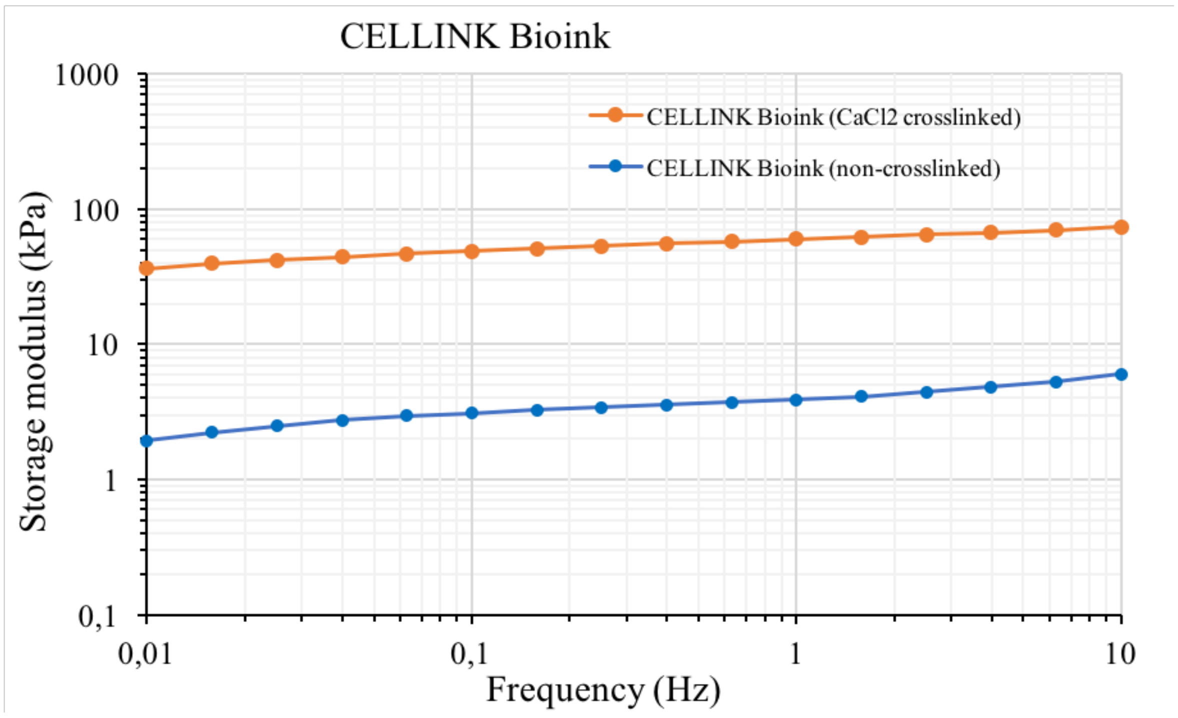

B

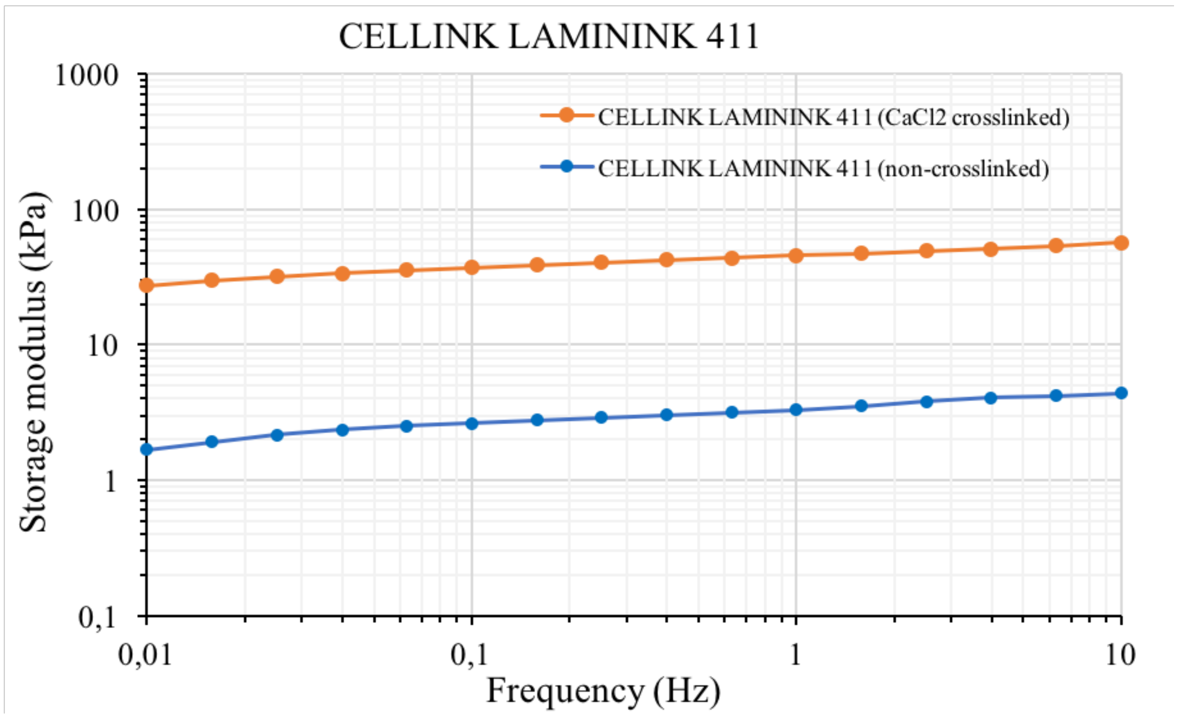

Supplementary figure 1. Cellink Bioink (**A**) and Cellink Laminink411 (**B**) rheology data regarding their stiffness, measured before (blue line) and after (orange line) crosslinking of 50 mM $CaCl_2$ for 30 minutes.

Supplementary figure 2

MEC-GPF inside **MEC-GPF outside**

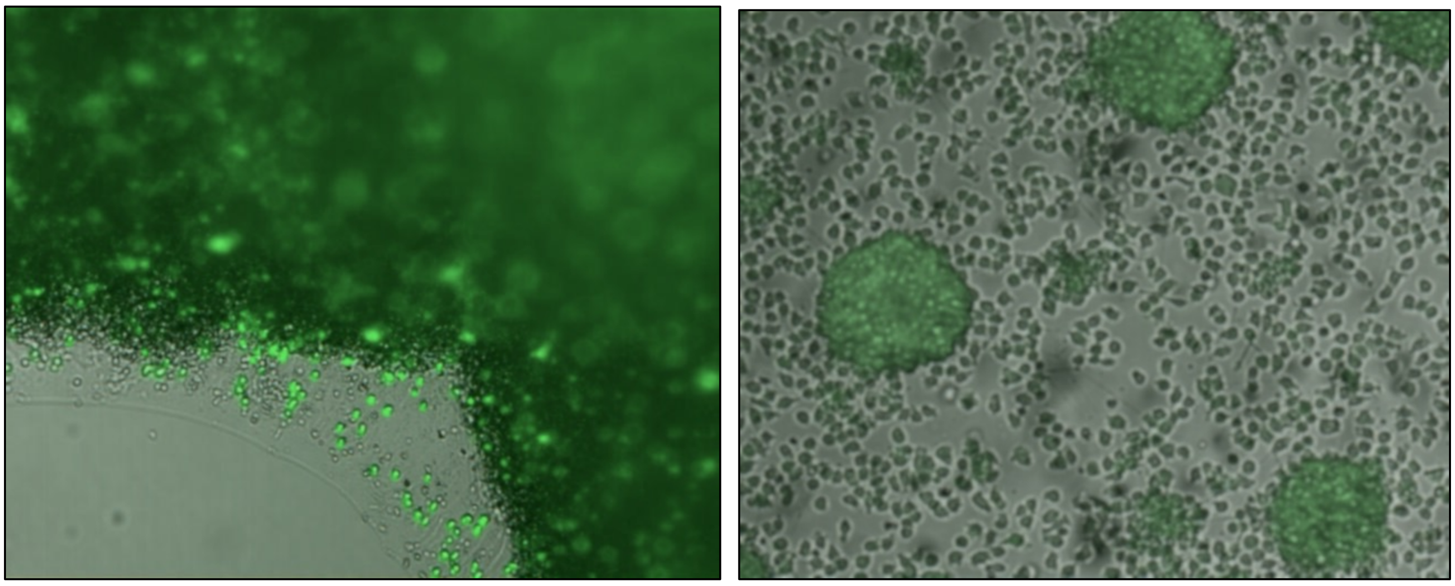

Day 14

Supplementary figure 2. Representative images of MEC-GFP cells inside and outside the 3D bioprinted scaffold, 14 days after printing. Images were acquired with JuLI™ Stage fluorescent microscope.

Supplementary figure 3

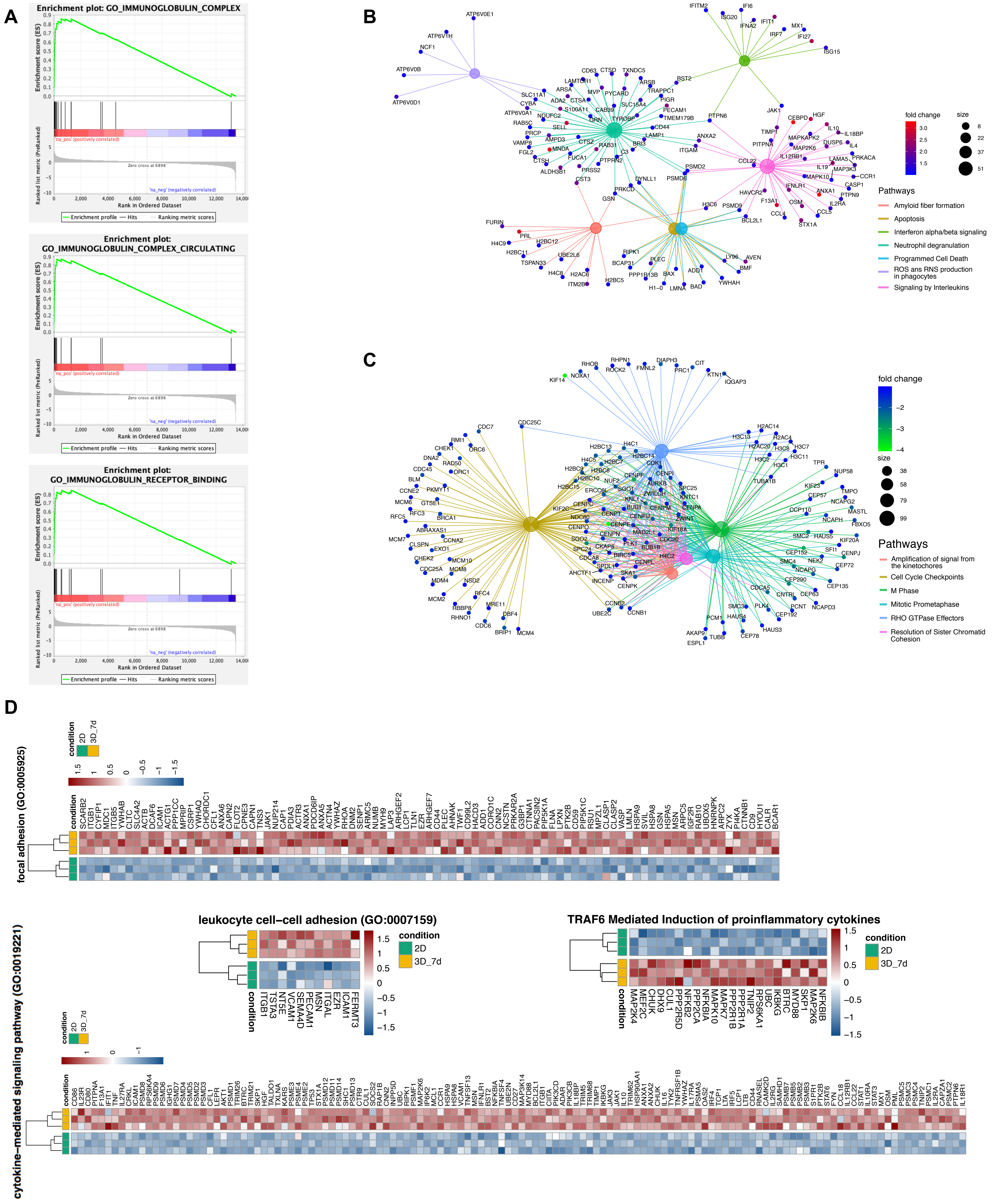

Supplementary figure 3. (**A**) GSEA curves for the first most significantly up-regulated pathways for Gene Ontology database in the comparison 3D_14d_vs_2D. (**B, C**) Cnetplots highlighting up-regulated (**B**) and down-regulated (**C**) pathways between the comparisons 3D_14d_vs_2D. (**D**) Heatmaps showing the most significantly modulated pathways in the comparison 3D_7d_vs_2D.

Supplementary figure 4

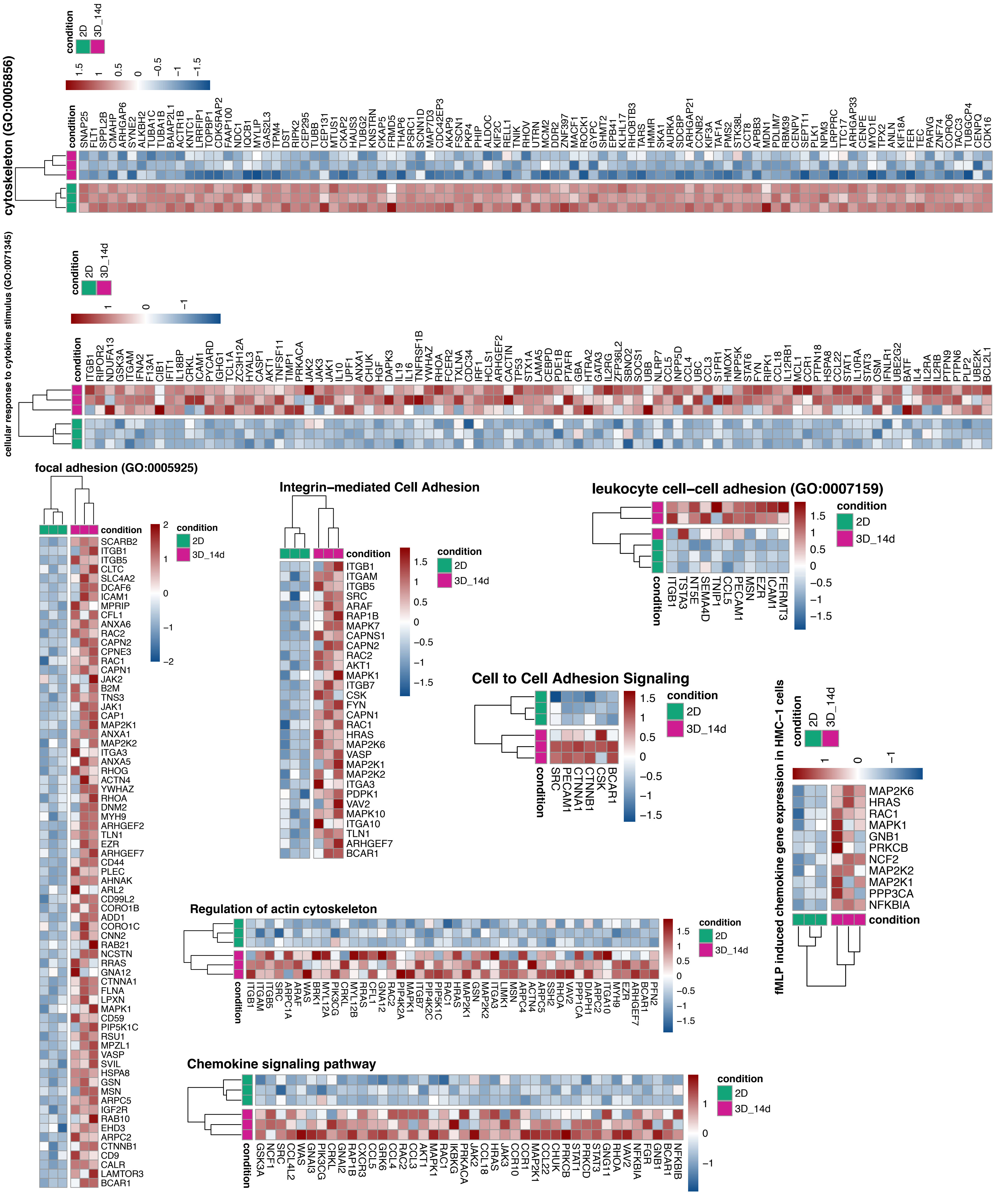

Supplementary figure 4. Heatmaps showing the most significantly modulated pathways in the comparison 3D_14d vs 2D.

Supplementary figure 5

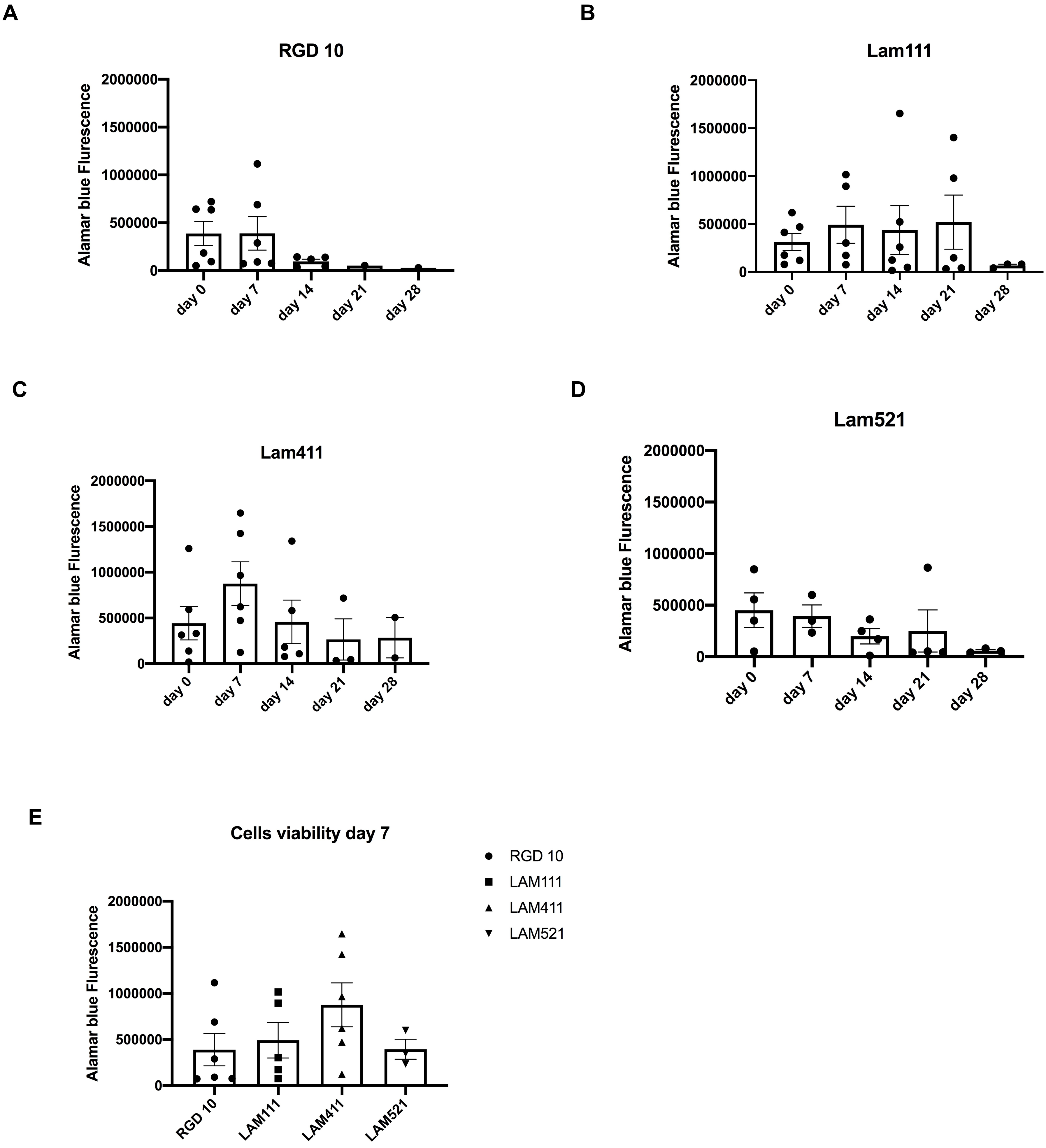

Supplementary figure 5. (**A**-**E**) The graphs show Alamar blue assay fluorescence values of 3D bioprinted CLL primary cells at different time points (day 0-7-14-21-28) by using different hydrogel matrices: (**A**) RGD 10 peptide, (**B**) Lamininink111, (**C**) Lamininink411, (**D**) Lamininink521. (**E**) The graph compares Alamar blue assay fluorescence values, after 7 days of culture, of CLL primary cells 3D bioprinted in different hydrogel matrices. Data are represented as mean±SEM, n=4 (**D**), n=6 (**A, B, C, E**) patient samples.

Supplementary figure 6

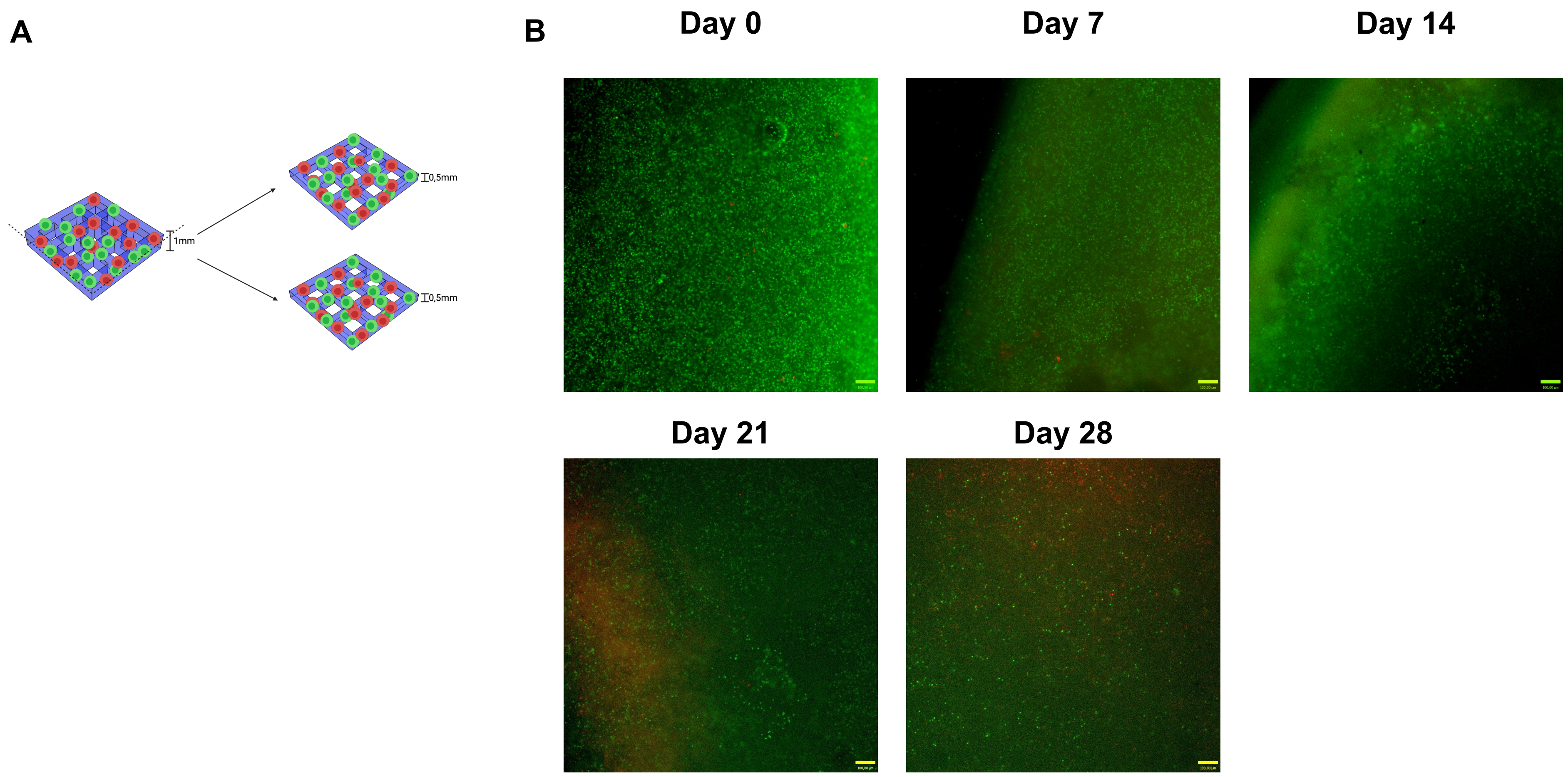

Supplementary figure 6. (**A**) Schematic representation of how Live/Dead viability assay was performed: after the scaffold has been treated with the kit reagents, it has been cut in two halves and then imaged at the Zeiss Axio Observer microscope. (**B**) Representative images of Live/Dead assay of 3D bioprinted CLL primary cells at different time points (day 0-7-14-21-28) acquired with Axio Observer Zeiss fluorescent microscope. Green cells are alive cells; red cells are dead cells.

Supplementary figure 7

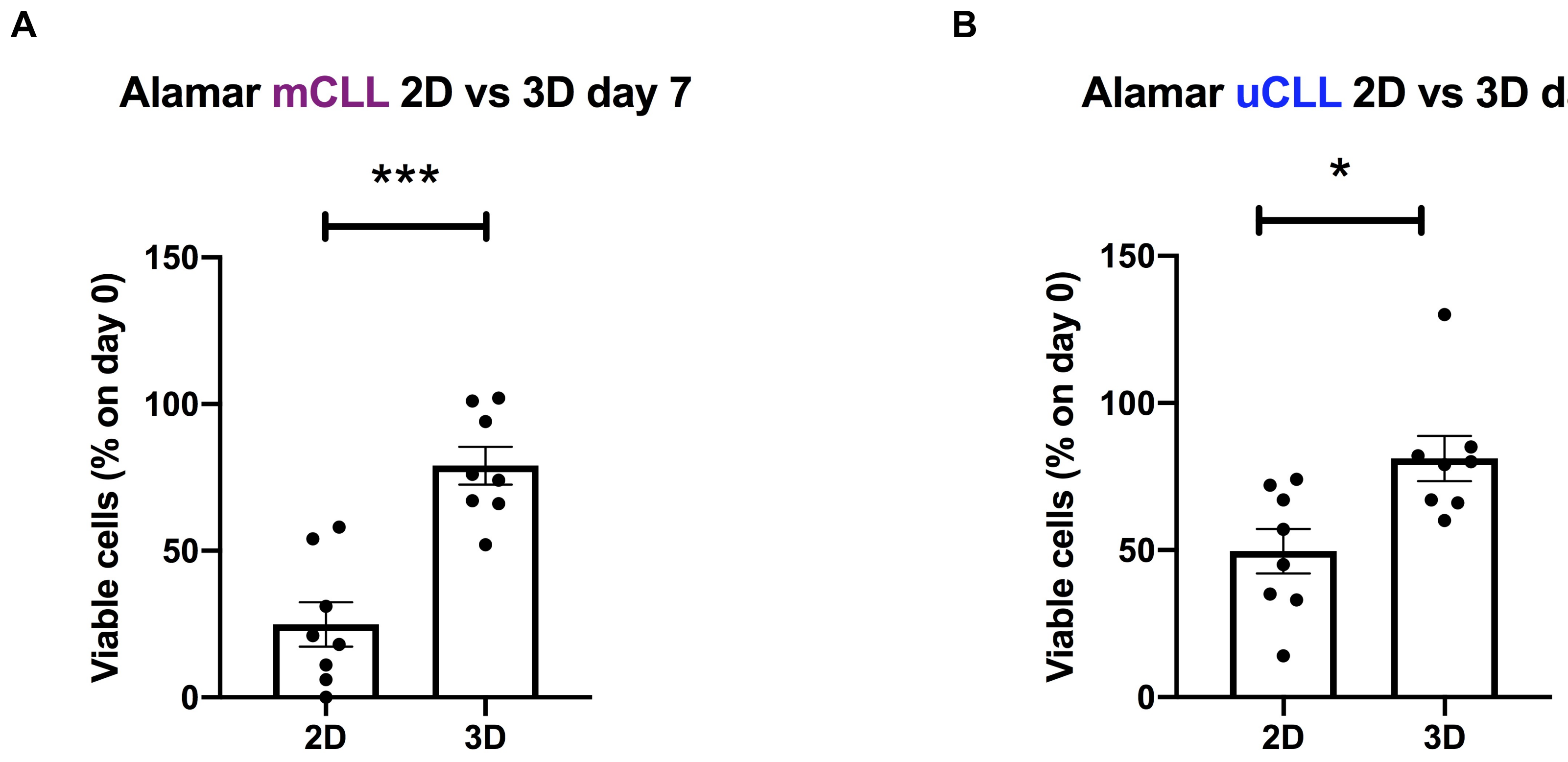

Supplementary figure 7. (**A**, **B**) The graphs show the percentages of viable cells normalized to day 0, measured by Alamar blue assay, after 7 days of culture, of 3D bioprinted CLL primary cells compared to CLL primary cells cultured in the traditional 2D system, of both (**A**) mCLL and (**B**) uCLL patients' groups of cells.

***p<0.001, *p<0.05. Data are represented as mean±SEM, n=8 patient samples. Mann-Whitney t-test was used for statistical analysis.

Supplementary figure 8

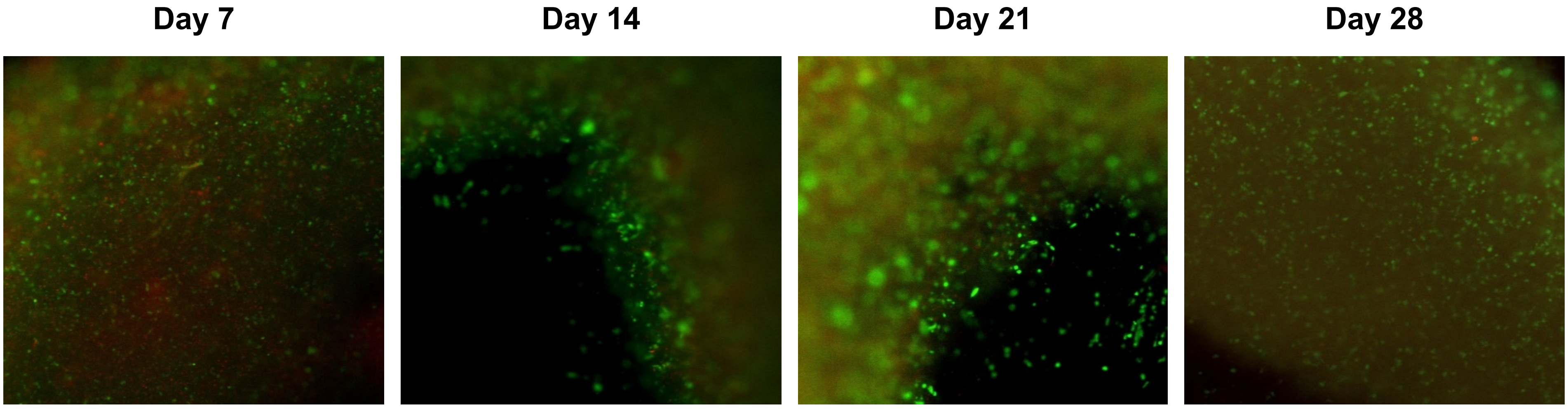

Supplementary figure 8. Representative images of Live/Dead assay of 3D bioprinted CLL primary PBMC cells at different time points (day 7-14-21-28) acquired with Axio Observer Zeiss fluorescent microscope. Green cells are alive cells; red cells are dead cells.

Supplementary figure 9

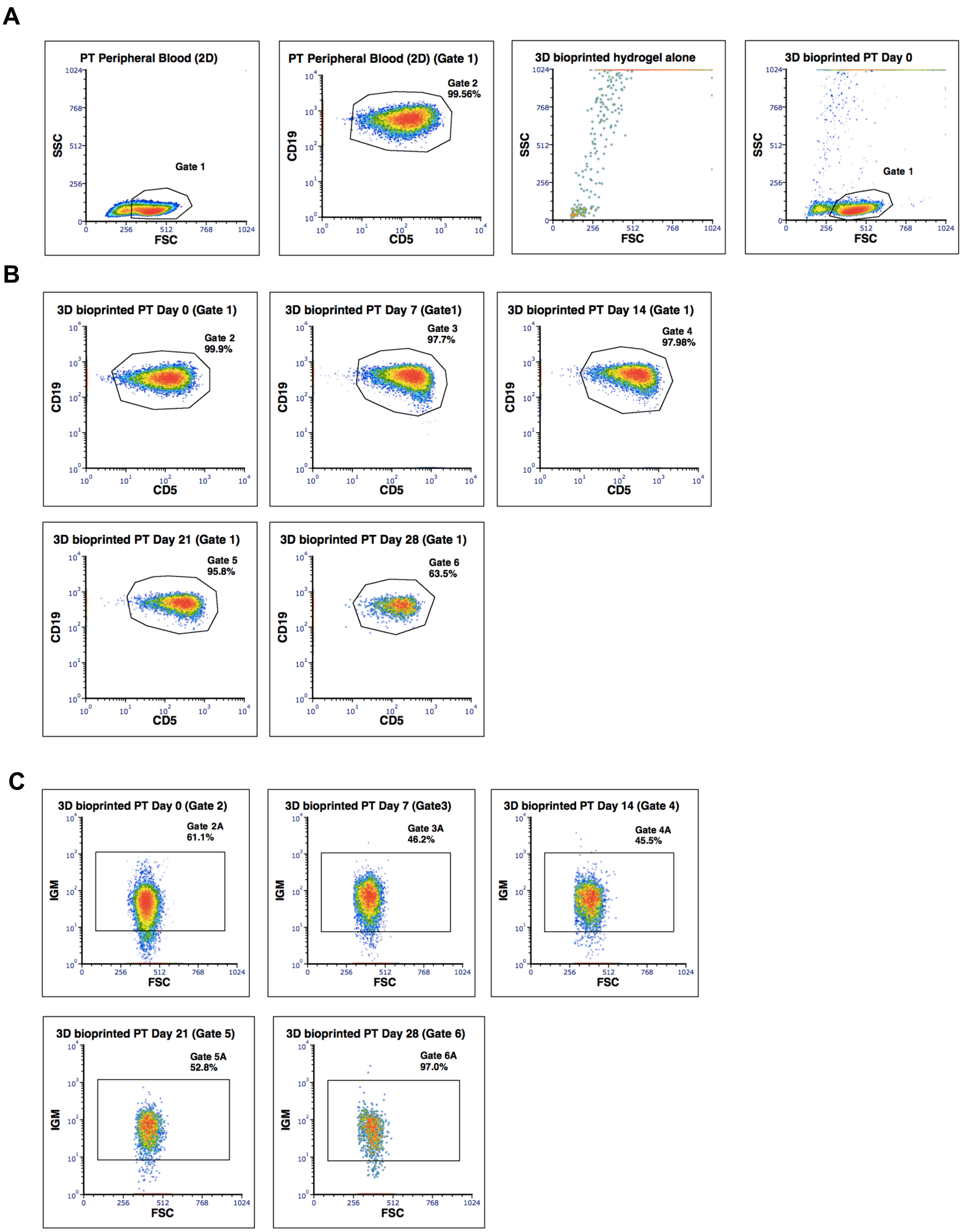

Supplementary figure 9. (**A**) Representative flow cytometry plots of 2D (left) and 3D (right) bioprinted CLL primary cells showing the presence of the leukemic clone, based on the expression of CD19 and CD5 surface markers. Physical parameters of 3D bioprinted hydrogel alone are shown as well. (**B**) Representative flow cytometry plots of 3D bioprinted CLL primary cells showing the presence of the leukemic clone overtime (day 0-7-14-21-28), based on the expression of CD19 and CD5 surface markers. (**C**) Representative flow cytometry plots of 3D bioprinted CLL primary cells showing the presence of IgM surface marker overtime.

Supplementary figure 10

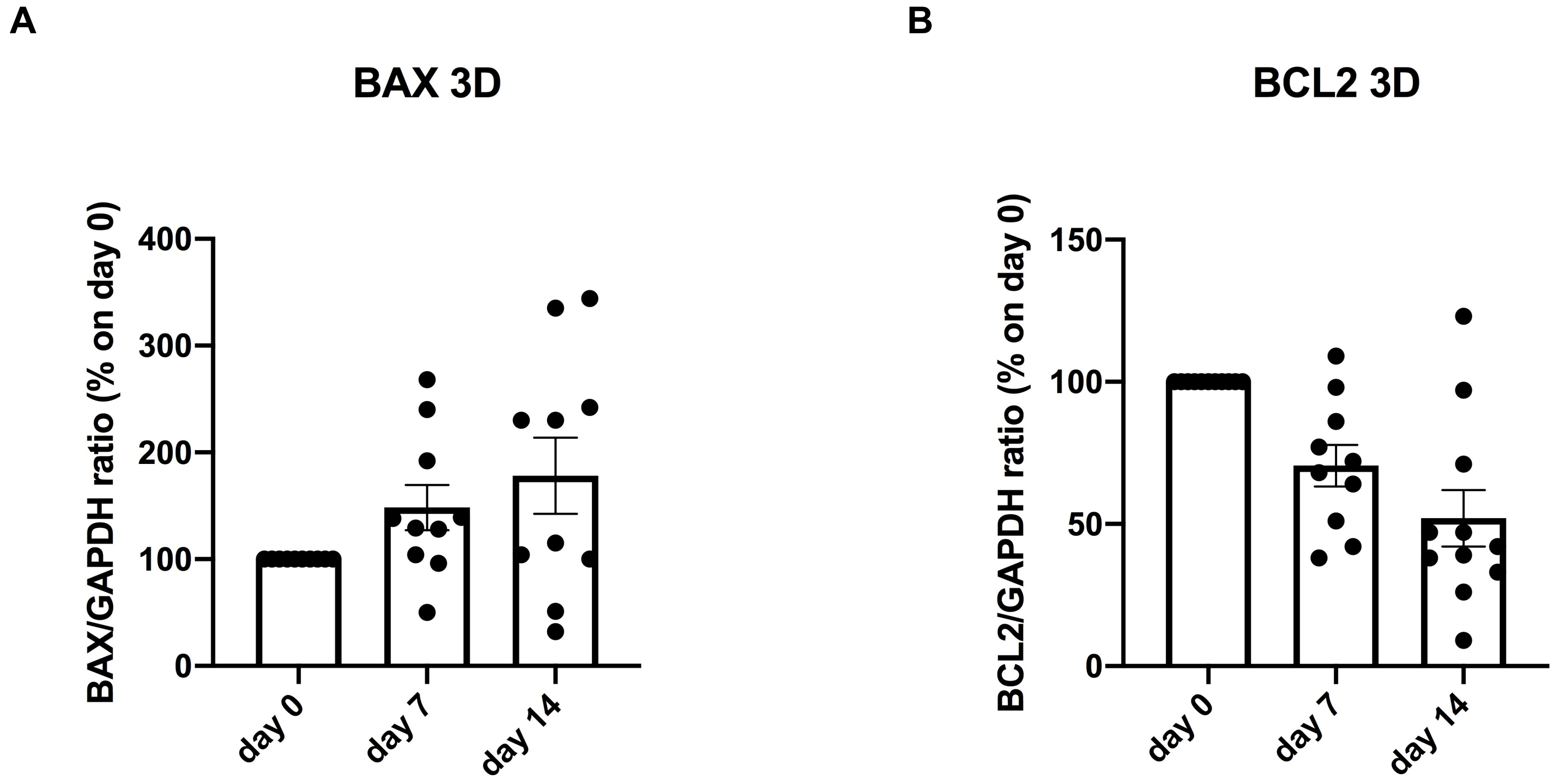

Supplementary figure 10. (**A**, **B**) The graphs show mRNA levels of, respectively, pro-apoptotic gene BAX and anti-apoptotic gene BCL2 of 3D bioprinted CLL primary cells at different time points (day 0-7-14). Data are represented as mean±SEM, n=10 patient samples.
